# Kv2 Ion Channels Determine the Expression and Localization of the Associated AMIGO-1 Cell Adhesion Molecule in Adult Brain Neurons

**DOI:** 10.3389/fnmol.2018.00001

**Published:** 2018-01-19

**Authors:** Hannah I. Bishop, Melanie M. Cobb, Michael Kirmiz, Laxmi K. Parajuli, Danielle Mandikian, Ashleigh M. Philp, Mikhail Melnik, Juha Kuja-Panula, Heikki Rauvala, Ryuichi Shigemoto, Karl D. Murray, James S. Trimmer

**Affiliations:** ^1^Department of Neurobiology, Physiology and Behavior, University of California, Davis, Davis, CA, United States; ^2^Center for Neuroscience, University of California, Davis, Davis, CA, United States; ^3^Division of Cerebral Structure, National Institute for Physiological Sciences, Okazaki, Japan; ^4^Neuroscience Center, University of Helsinki, Helsinki, Finland; ^5^Department Physiology and Membrane Biology, University of California, Davis, Davis, CA, United States

**Keywords:** ion channel, auxiliary subunit, brain, immunohistochemistry, cell adhesion molecule

## Abstract

Voltage-gated K^+^ (Kv) channels play important roles in regulating neuronal excitability. Kv channels comprise four principal α subunits, and transmembrane and/or cytoplasmic auxiliary subunits that modify diverse aspects of channel function. AMIGO-1, which mediates homophilic cell adhesion underlying neurite outgrowth and fasciculation during development, has recently been shown to be an auxiliary subunit of adult brain Kv2.1-containing Kv channels. We show that AMIGO-1 is extensively colocalized with both Kv2.1 and its paralog Kv2.2 in brain neurons across diverse mammals, and that in adult brain, there is no apparent population of AMIGO-1 outside of that colocalized with these Kv2 α subunits. AMIGO-1 is coclustered with Kv2 α subunits at specific plasma membrane (PM) sites associated with hypolemmal subsurface cisternae at neuronal ER:PM junctions. This distinct PM clustering of AMIGO-1 is not observed in brain neurons of mice lacking Kv2 α subunit expression. Moreover, in heterologous cells, coexpression of either Kv2.1 or Kv2.2 is sufficient to drive clustering of the otherwise uniformly expressed AMIGO-1. Kv2 α subunit coexpression also increases biosynthetic intracellular trafficking and PM expression of AMIGO-1 in heterologous cells, and analyses of Kv2.1 and Kv2.2 knockout mice show selective loss of AMIGO-1 expression and localization in neurons lacking the respective Kv2 α subunit. Together, these data suggest that in mammalian brain neurons, AMIGO-1 is exclusively associated with Kv2 α subunits, and that Kv2 α subunits are obligatory in determining the correct pattern of AMIGO-1 expression, PM trafficking and clustering.

## Introduction

Mammalian voltage-gated potassium (Kv) channels function as macromolecular assemblies of four voltage-sensing and pore-forming principal or α subunits (Trimmer, 2015). Substantial structural and functional heterogeneity in native Kv channels is generated from the combinatorial assembly of two dozen α subunit genes into homo- and hetero- tetrameric complexes, and their subsequent posttranslational modification (Trimmer, 2015). This already substantial diversity is further enhanced by coassembly with a large repertoire of cytoplasmic and/or transmembrane auxiliary subunits (Li et al., 2006; Pongs and Schwarz, 2010; Vacher and Trimmer, 2011). Kv channel auxiliary subunits impact Kv channel activity indirectly through effects on protein assembly and folding, regulation of biosynthetic intracellular trafficking, clustering at specific plasma membrane (PM) sites, and by influencing rates of endocytic recycling and degradation (Li et al., 2006; Pongs and Schwarz, 2010; Vacher and Trimmer, 2011). They can also exert profound effects on the function of Kv channels by impacting diverse aspects of channel gating (Li et al., 2006; Pongs and Schwarz, 2010; Vacher and Trimmer, 2011). This diversity provides opportunities for development of drugs and biologicals that impact channel function by modifying the interaction of auxiliary subunits with Kv channel complexes (Zhang et al., 2004; Lundby et al., 2010; Witzel et al., 2012; Ohya et al., 2016). Certain ion channel auxiliary subunits have been shown to exhibit functions independent of their role as auxiliary subunits, most notably the β subunits of voltage-gated sodium channels that function as cell adhesion molecules (Kruger and Isom, 2016).

Kv channels formed by the Kv2 α subunits Kv2.1 and Kv2.2 are highly expressed in mammalian brain neurons, where they exhibit robust clustering on cell bodies, proximal dendrites and the axon initial segment (Trimmer, 2015). Kv2.1 is widely expressed in mammalian brain where it is responsible for the majority of the delayed rectifier current in many neurons (Murakoshi and Trimmer, 1999; Du et al., 2000; Malin and Nerbonne, 2002; Bishop et al., 2015). Kv2.1 is highly phosphorylated (Misonou et al., 2006) and localized to PM clusters (Trimmer, 1991; Scannevin et al., 1996; Du et al., 1998; Mandikian et al., 2014; Bishop et al., 2015) that are found at endoplasmic reticulum: PM junctions (i.e., ER:PM junctions) (Du et al., 1998; Antonucci et al., 2001; Mandikian et al., 2014; Bishop et al., 2015; Fox et al., 2015). Kv2.1 phosphorylation is found primarily on the large cytoplasmic C-terminal tail (Trimmer and Misonou, 2015), which also contains a short motif required for clustering at ER: PM junctions (Lim et al., 2000; Fox et al., 2015). Kv2.2 differs from Kv2.1 in having a more restricted cellular expression in brain (Hwang et al., 1992), less extensive phosphorylation (Bishop et al., 2015; Trimmer and Misonou, 2015), and more constitutive clustering (Bishop et al., 2015) compared to Kv2.1. Kv2.1 and Kv2.2 can form heterotetrameric channels in coexpressing neurons (Kihira et al., 2010; Bishop et al., 2015), although certain brain neurons express high levels of Kv2.1 or Kv2.2 but not both (Hermanstyne et al., 2010; Bishop et al., 2015).

The single pass type I transmembrane protein AMIGO-1, which was originally discovered in a screen for genes induced by the neurite outgrowth promoting protein amphoterin (Kuja-Panula et al., 2003), was recently found to associate and colocalize with Kv2.1 in adult brain (Peltola et al., 2011). Unlike Kv2.1 and Kv2.2, AMIGO-1 has an extensive extracellular domain, which includes leucine-rich repeat and immunoglobulin domains. During development, AMIGO-1 acts as a homophilic cell adhesion molecule to promote neurite outgrowth in neurons (Kuja-Panula et al., 2003; Chen et al., 2012). AMIGO-1 colocalizes with Kv2.1 in large PM clusters in brain neurons both *in vivo* and *in vitro*, and when coexpressed in HEK293 cells (Peltola et al., 2011). Coexpression with AMIGO-1 produces a hyperpolarizing shift in the voltage-dependent gating of Kv2.1 (Peltola et al., 2011), and mice lacking AMIGO-1 expression have reduced Kv2.1 expression (Peltola et al., 2016), consistent with a role for AMIGO-1 as a *bona fide* auxiliary subunit of Kv2.1-containing channels. However, the full extent of AMIGO-1 association with the Kv2.1 and Kv2.2 α subunits in brain, and the role of Kv2 α subunits in determining the expression and localization of AMIGO-1, has not been investigated. Here, we use newly developed and KO-validated anti-AMIGO-1 antibodies (Abs) to define the expression and colocalization of AMIGO-1 with Kv2.1 and Kv2.2 in adult brain. We also analyze the impact of the Kv2 α subunits on expression and localization of AMIGO-1 in studies employing single and double Kv2.1 and Kv2.2 KO mice, and heterologous cells expressing WT and mutant Kv2 α subunits. These studies reveal an important role for Kv2 channels in supporting the expression and localization of AMIGO-1 in adult brain neurons.

## Materials and methods

Unless otherwise stated, all chemicals were from Sigma-Aldrich.

### Antibodies

Antibodies used here are listed in Table 1.

**Table 1:** Antibodies used in this study.

**Antibody name**	**Species/isotype/immunogen**	**Manufacturer information**	**Concentration used**
AMIGO-1, anti-AMIGO-1 rabbit pAb	Raised against a.a. 394-492 of mouse AMIGO-1 (cytoplasmic C-terminus)	Trimmer Lab. Rabbit 28330 RRID:AB_2571515	1:500 dilution of Affinity Purified pAb, concentration unknown
L86/33, anti-AMIGO-1 mouse IgG2a mAb	Raised against a.a. 394-492 of mouse AMIGO-1 (cytoplasmic C-terminus)	Trimmer lab. NeuroMab Cat# 73-317, RRID:AB_2315798	1:5 dilution of Tissue Culture Supernatant, concentration unknown
L86A/37, anti-AMIGO-1 mouse IgG2b mAb	Raised against a.a. 394-492 of mouse AMIGO-1 (cytoplasmic C-terminus)	Trimmer lab. NeuroMab Cat# 75-329, RRID:AB_2315801	1:5 dilution of Tissue Culture Supernatant, concentration unknown
L98/12, anti-AMIGO-1 mAb	Mouse IgG1 mAb, Raised against a.a. 28-370 of mouse AMIGO-1 (extracellular N-terminus)	Trimmer lab. RRID:AB_2571516	1:5 dilution of Tissue Culture Supernatant, concentration unknown
K89/34, anti-Kv2.1 mouse IgG1 mAb	Raised against a.a. 837-853 of rat Kv2.1	Trimmer lab. NeuroMab Cat# 73-014 RRID:AB_10672253	10 μg/mL Purified mAb
K89/34R, anti-Kv2.1 recombinant mouse IgG2a mAb	Raised against a.a. 837-853 of rat Kv2.1	Trimmer lab. Recombinant mouse mAb expressed in COS-1 cells. RRID:AB_2315768	1:2 dilution of Tissue Culture Supernatant, concentration unknown
N372B/1, anti-Kv2.2 mouse IgG1 mAb	Raised against a.a. 717-907 of rat Kv2.2. Binds within a.a. 764-907. Species reactivity with mouse, rat, ferret, macaque and human	NeuroMab. Cat# 73-369, RRID:AB_2315869	1:5 dilution of Tissue Culture Supernatant, concentration unknown
N372B/60, anti-Kv2.2 mouse IgG2b mAb	Raised against a.a. 717-907 of rat Kv2.2. Binds within a.a. 764-907. Species reactivity with mouse and rat	NeuroMab. Cat# 73-360, RRID:AB_2315867	1:5 dilution of Tissue Culture Supernatant, concentration unknown
25B6, anti-Ctip2 rat IgG2a mAb	Raised against aa 1-150 of human Ctip2	Abcam. Cat# ab18465, RRID:AB_2064130	0.5 μg/mL Purified mAb
M-222, anti-Cux1 rabbit pAb	Rabbit pAb, Raised against aa 1111-1332 of mouse Cux1/CDP	Santa Cruz Biotechnology. Cat# sc-13024, RRID:AB_2261231	0.4 μg/mL Purified pAb
SATBA4B10, anti-Satb2 mouse IgG1 mAb	Raised against recombinant fragment corresponding to C-terminus of human SATB2	Abcam. Cat# ab51502, RRID:AB_882455	0.1 μg/mL Purified mAb
N52A/42, anti-Mortalin/Grp75 mouse IgG1 mAb	Off target mAb from anti-Salm2 project	NeuroMab. Cat# 73-127, RRID:AB_10674108	1:20 dilution of Tissue Culture Supernatant, concentration unknown

*Details of the polyclonal (pAb) and monoclonal (mAb) Abs used in this study*.

### Generation of novel monoclonal and polyclonal antibodies

A hybridoma producing the L98/12 (IgG1) monoclonal antibody (mAb) was generated from a mouse immunized with extracellular N-terminus of AMIGO-1 using standard methods (Trimmer et al., 1985; Bekele-Arcuri et al., 1996). The immunogen was a Strep2-tagged fusion protein comprising the extracellular domain (a.a. 28–370) of AMIGO-1 produced in HEK293 cells (Kajander et al., 2011). The anti-AMIGO-1 rabbit polyclonal antibody (pAb) was generated at Pocono Rabbit Farm and Laboratory (Canadensis, PA) against amino acids 394–492 (cytoplasmic C-terminus) of mouse AMIGO-1, exhibiting 100% amino acid identity with rat and human AMIGO-1, and used previously to generate a chicken pAb (Peltola et al., 2011). Anti-AMIGO-1 polyclonal Abs were affinity purified from serum on nitrocellulose strips containing the immunogen following the method of Olmsted (Olmsted, 1981).

### Antibody characterization

Table 1 contains a list of all Abs used in this study.

The anti-AMIGO-1 affinity-purified rabbit pAb “AMIGO-1” (Trimmer Lab, RRID:AB_2571515) was validated by immunoblot against wild-type (WT) and AMIGO-1-KO mouse brain samples, by immunohistochemistry against WT and AMIGO-1-KO mouse brain sections (see below), and does not detect other AMIGO isoforms by immunocytochemistry and immunoblotting using samples from heterologous cells expressing either AMIGO-1, AMIGO-2, or AMIGO-3.

The anti-AMIGO-1 mouse mAbs L86/33 (NeuroMab Cat# 73-317, RRID:AB_2315798) and L86A/37 (NeuroMab Cat# 73-329, RRID:AB_2315765) were validated by immunoblot against WT and AMIGO-1-KO brain samples, and by immunohistochemistry against WT and AMIGO-1-KO mouse brain sections (see below). These mAbs do not detect other AMIGO isoforms on immunoblots of cell lysates prepared from heterologous cells expressing AMIGO-1, AMIGO-2, or AMIGO-3. The anti-AMIGO-1 mouse mAb L98/12 (Trimmer Lab, RRID:AB_2571516) was validated by immunohistochemistry against WT and AMIGO-1-KO mouse brain sections (see below), and against heterologous cells expressing AMIGO-1 and untransfected cells by ELISA and immunocytochemistry.

The anti-Kv2.1 K89/34 mouse mAb (NeuroMab Cat# 73-014, RRID:AB_10672253) was validated by immunoblot against WT and Kv2.1-KO mouse brain samples, and by immunohistochemistry against WT and Kv2.1-KO mouse brain sections, as described (Mandikian et al., 2014; Bishop et al., 2015). The anti-Kv2.1 K89/34R recombinant mouse mAb (Trimmer Lab, RRID:AB_2315768), which is the recombinant version of K89/34 with the IgG1 heavy chain constant region replaced with that of the IgG2a subclass (Mandikian et al., 2014) was validated by immunohistochemistry against WT and Kv2.1-KO mouse brain sections, as described (Mandikian et al., 2014).

The anti-Kv2.2 mouse mAbs N372B/1 (NeuroMab Cat# 73-369, RRID:AB_2315869) and N372B/60 (NeuroMab Cat# 73-360, RRID:AB_2315867) were validated by immunoblot against WT and Kv2.2-KO mouse brain samples, and by immunohistochemistry against WT and Kv2.2-KO mouse brain sections, as described (Bishop et al., 2015).

The anti-Ctip2 rat mAb 25B6 (Abcam Cat# ab18465, RRID:AB_2064130) was made against a fusion protein containing a.a. 1–150 of human Ctip2 and was validated by the manufacturer for use in immunohistochemistry, immunocytochemistry, immunoblot, immunoprecipitation, and chromatin immunoprecipitation analyses.

The anti-Cux1 rabbit pAb M-222 (Santa Cruz Biotechnology Cat# sc-13024, RRID:AB_2261231) was made against C-terminal a.a. 1,111–1,332 of mouse CDP (Cux1) and was validated by the manufacturer for use in immunohistochemistry, immunocytochemistry, immunoblot, immunoprecipitation, and chromatin immunoprecipitation analyses.

The anti-Satb2 mouse mAb SATBA4B10 (Abcam Cat# ab51502, RRID:AB_882455) was made against a recombinant C-terminal fragment of human Satb2 and was validated by the manufacturer for use in immunohistochemistry, immunocytochemistry, immunoblot, immunoprecipitation and chromatin immunoprecipitation analyses.

The anti-Mortalin/GRP75 mouse mAb N52A/42 (NeuroMab Cat# 73-127, RRID:AB_10674108) was validated by LC-MS/MS analysis of immunoprecipitation reactions performed against rat brain extracts, and by immunoblot against HEK293 cells expressing endogenous Mortalin/GRP75 using siRNA-mediated knockdown (manufacturer's specifications).

### Animals

All animal use procedures involving rats and Kv2 KO mice were performed in strict accordance with the Guide for the Care and Use of Laboratory Animals of the U.S. National Institutes of Health (NIH), and were approved by the University of California Davis Institutional Animal Care and Use Committee. Mice and rats were maintained under standard light–dark cycles and allowed to feed and drink *ad libitum*. Kv2.1-KO mice (RRID:IMSR_MGI:3806050) have been described previously (Jacobson et al., 2007; Speca et al., 2014), and were generated by breeding Kv2.1^+/−^ mice backcrossed onto a C57/BL6J background (RRID:IMSR_JAX:000664) such that all experiments with Kv2.1-KO mice used WT littermates as controls. Kv2.2-KO mice were obtained from Drs. Tracey Hermanstyne and Jeanne Nerbonne, and have been described previously (Hermanstyne et al., 2010, 2013). All Kv2.2^−/−^ mice used here were obtained from homozygotic crosses. Kv2.1 and Kv2.2 double-KO (Kv2 dKO) mice (Kv2.1^−/−^/Kv2.2^−/−^) were generated by breeding Kv2.1^+/−^ mice with Kv2.2^−/−^ mice. Both male and female mice were used, all over 10 weeks old. Sprague-Dawley rats (Female 200-300 g, Charles River) were >15 week old females.

### Preparation of brain sections

For preparation of brain sections, rats and mice were deeply anesthetized with 60 mg/kg sodium pentobarbital and transcardially perfused with ≈5 mL (mouse) or ≈20 mL (rat) phosphate-buffered saline (PBS; 150 mM NaCl, 10 mM sodium phosphate buffer, pH7.4) containing 10 U/mL heparin, followed by ≈30 mL (mouse) or ≈150 mL (rat) ice-cold 4% formaldehyde (freshly prepared from paraformaldehyde) in 0.1 M sodium phosphate buffer, pH 7.4 (0.1 M PB). The brains were removed and cryoprotected for 24 h in 10% sucrose 0.1 M PB, and then for 24–48 h in 30% sucrose in 0.1 M PB. Perfusion-fixed brains from age- and sex-matched WT and AMIGO-1 KO mice (Peltola et al., 2016) were prepared in strict accordance with the Guide for the Care and Use of Laboratory Animals of the U.S. National Institutes of Health (NIH), and were approved by the Helsinki University Institutional Animal Care and Use Committee. Perfusion-fixed and cryoprotected ferret brains were gifts from the laboratory of our late colleague Dr. Barbara Chapman. Fresh-frozen macaque samples were a gift from the laboratory of our late colleague Dr. Edward G. Jones. Fresh-frozen human brain samples (49.5-year-old Caucasian male, 5-h post-mortem interval) were obtained from the U.S. National Institute of Child Health and Human Development (NICHD) Brain and Tissue Bank for Developmental Disorders (NICHD contract HHSN275200900011C, ref. NO1-HD-9–0011). Samples from the visual cortex of human and macaque were thawed in the same fixative used for perfusions, fixed for 45 min at 4°C, and cryoprotected as for perfusion fixed brains. Following cryoprotection, all samples were frozen, and 30 μm sections were generated using a freezing stage sliding microtome (Richard Allen Scientific HM 450). Sections were collected in 0.1 M PB and processed for immunohistochemistry.

### Multiplex immunofluorescence labeling of brain sections

Multiplex immunofluorescence labeling was performed essentially as described previously (Manning et al., 2012; Bishop et al., 2015). In brief, free-floating sections were incubated in 10% goat serum in 0.1 M PB containing 0.3% Triton X-100 (vehicle) for 1 h and then incubated 3 h at room temperature (RT) in vehicle containing different combinations of primary Abs, as detailed in the respective figure legends. Following incubation, sections were washed three times for 10 min each in 0.1 M PB, and incubated for 1 h in vehicle containing affinity-purified species and/or mouse IgG–subclass-specific goat secondary Abs (Manning et al., 2012) conjugated to Alexa Fluors (Molecular Probes/ThermoFisher). Sections were labeled with the DNA-specific dye Hoechst 33258 during the secondary antibody step. Following three 10 min washes in 0.1 M PB, sections were mounted and dried onto gelatin-coated slides, treated with 0.05% Sudan Black (EM Sciences) in 70% ethanol for 5 min (Schnell et al., 1999), extensively washed in water, and mounted with Prolong Gold (Life Technologies/ThermoFisher).

Low-magnification images were acquired on a Zeiss AxioObserver Z1 microscope using a 10 × /0.5 NA Fluar objective and an AxioCam HRm digital camera, and reconstructed as tiled mosaics using Axiovision 4.0 (Carl Zeiss MicroImaging, RRID:S- ciRes_000111). High-magnification images were acquired on a Zeiss AxioImager M2 microscope using a 40 × /0.8 NA plan-Apochromat oil-immersion objective, or a 63 × /1.40 NA plan-Apochromat oil immersion objective, and an AxioCam MRm digital camera. Optical sections were acquired using an ApoTome 2 structured illumination system (Carl Zeiss MicroImaging). Imaging and post processing was performed in Axiovision (Carl Zeiss MicroImaging), ImageJ (National Institutes of Health) and MATLAB (MathWorks). Super-resolution light microscopy was performed on a Zeiss Elyra system (SR-SIM) using a 100 × /1.4 NA plan-Apochromat oil immersion objective and Z stacks were reconstructed with Zen software (Carl Zeiss MicroImaging). Linear adjustments to contrast and brightness were performed using Photoshop (Adobe Systems) or ImageJ. All panels in a given figure were imaged and treated identically, unless otherwise noted.

### Electron microscopic immunohistochemistry

All mouse handling and sample preparation procedures for electron microscopy were done at the National Institute for Physiological Sciences, Okazaki, Japan and were conducted under regulatory guidelines of the institution. Adult mice were anesthetized with pentobarbital and transcardially perfused with 100 ml of ice-cold 4% formaldehyde (freshly prepared from paraformaldehyde) in 15% saturated picric acid/0.05% glutaraldehyde in 0.1 M PB, pH 7.4. Brains were removed and sectioned to 50 μm using a vibratome (Leica model VT-1000). Sections were incubated in a blocking solution containing 20% normal goat serum in 50 mM Tris (pH 7.6) plus 150 mM NaCl for 1 h. Sections were then incubated for 48 h at 4°C with purified anti-Kv2.1 K89/34 mouse mAb (diluted to 10 μg/mL in vehicle: 2% NGS in TBS) or anti-AMIGO-1 L86/33 mouse mAb tissue culture supernatant (diluted 1:2 in vehicle). After three washes in 25 mM PB (pH 7.4) plus 150 mM NaCl (25 mM PBS), sections were incubated overnight at 4°C with an anti-mouse secondary antibody conjugated to 1.4 nm gold particles (Nanoprobes, 1:100 dilution). Sections were washed three times in 25 mM PBS and once in milliQ water. Thereafter, immunogold particles were silver intensified using the HQ silver enhancement kit (Nanoprobes). Silver intensified sections were treated with 1% osmium tetroxide, *en bloc* counterstained with uranyl acetate, dehydrated and flat embedded in Durcupan resin (ACM Fluka, Sigma-Aldrich). Ultrathin sections (70 nm) were collected on formvar coated single-slot copper grids, counterstained briefly with freshly prepared 1% lead citrate and analyzed using a Philips transmission electron microscope (EM208S) equipped with a MegaView III CCD camera (Olympus-SIS).

### Heterologous cell culture and transfection

HEK293 cells were maintained in Dulbecco's modified Eagle's medium supplemented with 10% Fetal Clone III (HyClone), 1% penicillin/streptomycin, and 1X GlutaMAX (ThermoFisher). HEK293 cells were split to 15% confluence then transiently transfected 24 h later with the respective plasmids. These included plasmids encoding rat Kv2.1 (Frech et al., 1989; Shi et al., 1994) or the non-clustering rat Kv2.1 mutant S586A (Lim et al., 2000), and/or rat Kv2.2 (Kihira et al., 2010), or the non-clustering rat Kv2.2 mutant S605A (Bishop et al., 2015), all in the mammalian expression vector pRBG4 (Lee et al., 1991) and/or mouse AMIGO-1 in the mammalian expression vector PC DNA6 V5 His Version A (Peltola et al., 2011). Transfections were performed using LipofectAMINE 2000 (Invitrogen/ThermoFisher) transfection reagent following the manufacturer's protocol. HEK293 cells were transfected in DMEM without supplements, then returned to regular growth media 4 h after transfection. For live cell imaging experiments, HEK293 cells were transiently transfected with the general ER marker SEC61β-BFP, and DsRed-Kv2.1 and/or YFP-AMIGO-1 using the same approach. YFP-AMIGO-1 for live cell imaging was generated *via* Gibson assembly of mouse AMIGO-1 into the YFP-N1 vector (ClonTech) resulting in fusion of YFP to the cytoplasmic C-terminus of full-length mouse AMIGO-1. DsRed-Kv2.1 was generated *via* insertion of full-length rat Kv2.1 into the DsRed-C1 vector (ClonTech) resulting in fusion of DsRed to the N-terminus of full-length Kv2.1. The BFP-Sec61 beta plasmid encoding SEC61β-BFP (Zurek et al., 2011) was a gift from Gia Voeltz (Addgene plasmid # 49154). All plasmids were verified by sequencing. HEK293 cells were used 40–48 h post-transfection. Cells were grown in a humidified incubator at 37°C and 5% CO_2_.

### Immunofluorescence labeling of HEK293 cells

Immunofluorescence labeling was performed on permeabilized (Shi et al., 1994; Cobb et al., 2015) or intact (Shi et al., 1996) fixed cells essentially as described. Briefly, labeling of fixed and permeabilized cells was performed on HEK293 cells grown on poly-L-lysine coated glass coverslips, and fixed for 15 min at 4°C in ice-cold fixation buffer containing 4% formaldehyde (prepared fresh from paraformaldehyde), 4% sucrose, 0.1% Triton X-100 in DPBS (137 mM NaCl, 2.7 mM KCl, 10 mM Na_2_HPO_4_, 1.7 mM KH_2_PO_4_, pH 7.4) containing 1 mM CaCl_2_ and 1 mM MgCl_2_. All subsequent steps were performed at RT. Cells were washed 3X for 10 min each in PBS, blocked for 45 min with Blotto (3% (w/v) non-fat dry milk powder in TBS) with 0.1% Triton X-100, then incubated in Blotto containing primary Abs for 1 h. For cell surface labeling of intact HEK293 cells, cells were fixed for 25 min 4°C in the same fixative as above minus the Triton X-100. All subsequent steps were performed at RT. Fixed cells were washed 3X for 10 min each in PBS, blocked with Blotto without Triton X-100 for 45 min, incubated with anti-AMIGO-1 mouse mAb L98/12 diluted in Blotto without Triton X-100 for 1 h, washed 3X for 10 min each in PBS, then permeabilized for 30 min with Blotto containing 0.1% Triton X-100, followed by incubation with anti-AMIGO-1 rabbit pAb and/or Kv2.1 and Kv2.2 mAbs for 1 h. Primary Abs were detected with goat secondary Abs against either the specific mouse IgG subclass of the mAbs, or against rabbit IgG, conjugated to Alexa Fluors (Molecular Probes/ThermoFisher). Cells were labeled with Hoechst 33258 during incubation with secondary Abs. Images were acquired with an AxioCam MRm digital camera installed on a Zeiss AxioImager M2 microscope, or with an AxioCam HRm digital camera installed on a Zeiss AxioObserver Z1 microscope, using a 63X/1.40 NA plan-Apochromat oil immersion objective and an ApoTome coupled to Axiovision software (Carl Zeiss MicroImaging).

### Live cell TIRF imaging of HEK293 cells

Total internal reflection fluorescence (TIRF) imaging was performed at the MCB Imaging Facility at UC Davis. Live transfected HEK293 cells were imaged in a physiological saline solution (4.7 mM KCl, 146 mM NaCl, 2.5 mM CaCl2, 0.6 mM MgSO4, 1.6 mM NaHCO3. 0.15 mM NaH2PO4, 20 mM HEPES, pH 7.4) containing 8 mM glucose and 0.1 mM ascorbic acid. Cells were maintained at 37°C during the course of imaging with a heated stage and objective heater. Images were obtained with an Andor iXon EMCCD camera installed on a TIRF/widefield equipped Nikon Eclipse Ti microscope using a Nikon LUA4 laser launch with 405, 488, 561, and 647 nm lasers and a 100X PlanApo TIRF, 1.49 NA objective run with NIS Elements software (Nikon). Images of SEC61β-BFP, YFP-AMIGO-1 and DsRed-Kv2.1 were acquired at a constant exposure time in TIRF.

### Image analysis

All images were transferred to PhotoShop software (Adobe Systems) or ImageJ (National Institutes of Health) as linear 8-bit TIF files. For clustering analyses in heterologous cells, an *n* of four independent samples of at least 100 cells each was scored for having clustered or dispersed localization. For analysis of clustering in heterologous HEK293 cells by coefficient of variation, ImageJ was used to measure the mean and standard deviation of the pixel intensity values of regions of interest (ROIs) drawn around each cell, excluding the edge of the cell, from an *n* of four independent samples of at least 14 cells each, imaged with a 63X objective. For analysis of clustering by coefficient of variation in brain sections, at least 25 cells per section from three independent animals were analyzed. For AMIGO-1 cell surface expression analysis, the mean pixel intensity values of cell surface AMIGO-1 (mAb L98/12) immunolabeling and total AMIGO-1 (AMIGO-1 rabbit pAb) immunolabeling of ROIs drawn around each cell, excluding the edge of the cell, were measured using ImageJ. The ratio of cell surface to total AMIGO-1 immunolabeling was calculated and normalized to the average ratio in cells expressing AMIGO-1 alone for that independent sample. This analysis was performed in four independent samples of at least an *n* = 67 cells total, imaged with a 63X objective. All images were measured from Z stacks of optical sections taken at equal exposure across all images within an experiment. Representative images were taken with optimal exposure time and the brightness and contrast linearly adjusted to optimize display.

Analysis of colocalization between SEC61β-BFP, YFP-AMIGO-1, and DsRed-Kv2.1 was performed in NIS elements. A region of interest was drawn within each cell analyzed, excluding the edge of the cell, and Pearson's correlation coefficients were collected. Quantification of ER:PM junction size was performed in FIJI. Images (ND2 files) of BFP-SEC61β collected in TIRF were imported directly into FIJI, background subtracted using a rolling ball radius of 10 pixels, converted into an 8 bit image, and automatically converted into a binary mask using auto local thresholding. An ROI with identical dimensions was drawn within each cell analyzed. The average size of ER:PM junctions was quantified automatically using the “analyze particles” function within FIJI. ER:PM junctions smaller than 0.04 μm^2^ were excluded from this analysis. These data sets were imported into GraphPad Prism software for presentation and statistical analysis. Means are shown ± standard deviation.

### Proteinase K digestion of HEK293 cells

Proteinase K analysis of cell surface expression was performed essentially as described (Zhou et al., 1998; Manganas and Trimmer, 2000). Briefly, live transfected HEK293 cells were washed once at 37°C with DPBS with 1 mM CaCl_2_ and 1 mM MgCl_2_, followed by 30 min incubation at 37°C with 10 mM HEPES, 150 mM NaCl, and 2 mM CaCl_2_ (pH 7.4) with or without 200 μg/mL Proteinase K (Roche). The reaction was quenched by adding ice-cold DPBS (without 1 mM CaCl_2_ and 1 mM MgCl_2_) containing 6 mM phenylmethylsulfonyl fluoride (PMSF) and 25 mM EDTA. Cells were harvested with a cell scraper, pelleted by centrifugation at 4°C at 400 × g for 5 min in a microcentrifuge, and resuspended in reducing SDS sample buffer. Lysates were analyzed by immunoblot as described below.

### Preparation of crude mouse brain homogenates

For preparation of crude brain homogenates, mice were decapitated without anesthesia and brains were removed and homogenized within a 1 min post-mortem period in homogenization buffer (5 mM sodium phosphate, pH 7.4, 320 mM sucrose, 100 mM NaF, 500 μM PMSF and a protease inhibitor mixture (2 μg/mL aprotinin, 2 μg/mL anti-pain, 1 μg/mL leupeptin, and 10 μg/mL benzamidine). Protein concentration was determined by BCA assay (Pierce/ThermoFisher).

### SDS-PAGE and immunoblots

Cell lysates were prepared from transfected HEK293 cells as described (Shi et al., 1994; Manganas and Trimmer, 2000). In brief, cells were lysed for 5 min on ice in an ice-cold lysis buffer solution containing TBS (20 mM Tris, 150 mM NaCl, pH 8.0), 10 mM EDTA, 2% Triton X-100, 10 mM iodoacetamide, 10 mM NaF, and a protease inhibitor mixture (2 μg/mL aprotinin, 1 μg/mL leupeptin, 2 μg/mL antipain, 10 μg/mL benzamidine, and 1 mM PMSF). The detergent lysate was centrifuged in a microcentrifuge for 5 min at 16,100 × g to pellet nuclei and debris, and the resulting supernatant (cleared lysate) was saved for analysis. For immunoblots, crude brain homogenate or MBM fractions (30 μg protein/lane) or the HEK293 cell cleared lysate was added to one-third volume of 4X reducing SDS sample buffer and fractionated on 9% SDS-polyacrylamide gels, and transferred to nitrocellulose membranes. All subsequent steps were performed at RT. Immunoblots were blocked in 4% non-fat dry milk/0.1% Tween-20/TBS, and probed with various Abs. Primary Abs were detected with mouse IgG subclass-specific rabbit or goat secondary Abs conjugated to Alexa Fluors (Molecular Probes/ThermoFisher) using a FluorChem Q imager (ProteinSimple). Immunoblots were analyzed using FluorChem software and statistical analysis was performed using StatPlus or Prism. Note that “lauryl sulfate” (Sigma L-5750; 69% lauryl sulfate (SDS), 26% myristyl sulfate, 5% cetyl sulfate) was used in SDS gel recipes to accentuate electrophoretic mobility differences between different phosphorylation states of Kv2.1 (Shi et al., 1994; Murakoshi et al., 1997) and Kv2.2 (Bishop et al., 2015).

### Statistics

Prism (GraphPad) software was used to perform statistical tests on the data obtained from the cortical neurons and HEK cells. For Manders' colocalization coefficient (MCC) measurements, a standard one-way ANOVA with post hoc Sidak's multiple-comparison test was used to determine which MCC averages differed. For Coefficient of Variation (CV) measurements, a one-way randomized block ANOVA (experiments were treated as matched sets to account for experiment-to-experiment variability) with post hoc Sidak's multiple-comparison test was used to determine which individual CV means differed and summary data are presented as across-replicate means ± SEM.

## Results

### Anti-AMIGO-1 antibodies exhibit specific immunoreactivity in immunohistochemistry assays on mouse brain samples

**Figure 1:**
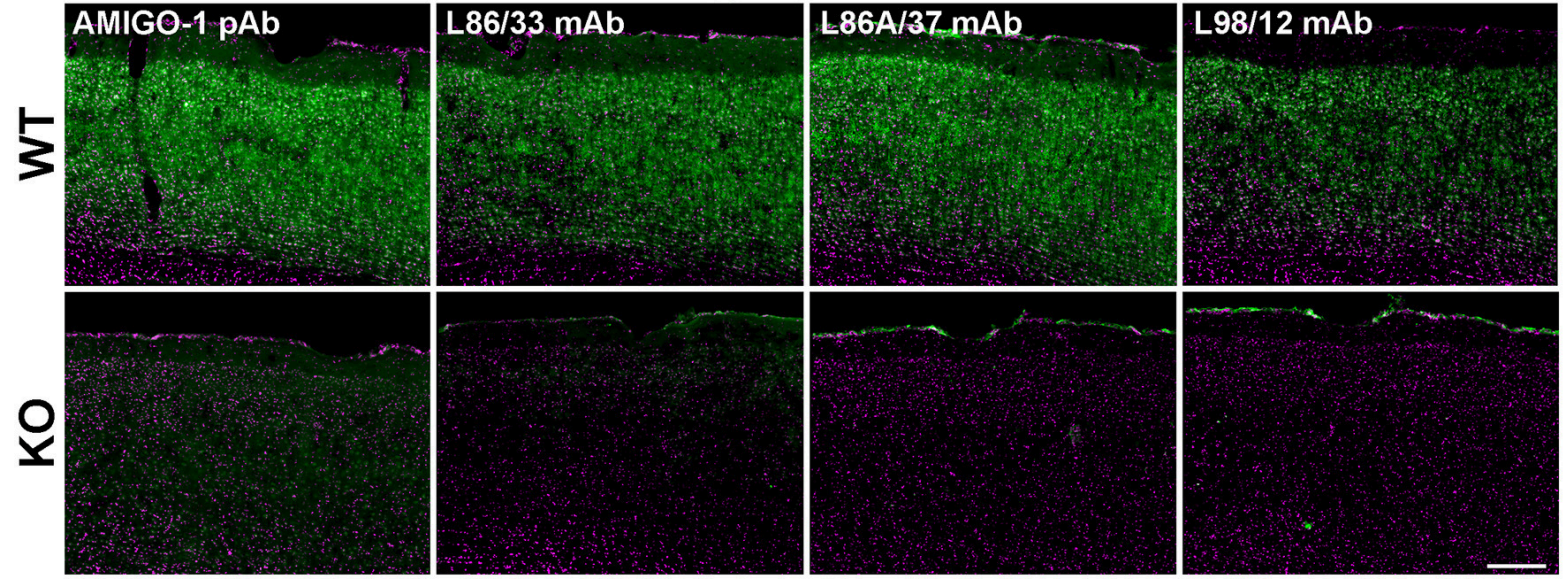
Validation of anti-AMIGO-1 antibodies in WT and KO mouse brain. Images of brain sections prepared from WT **(Upper)** and KO **(Lower)** mice, and immunolabeled and imaged under identical conditions. Green = anti-AMIGO-1 immunolabeling. Magenta = Hoechst 33258 nuclear dye. Antibodies used as indicated. Scale bar = 200 μm.

A previously published study presented images of immunofluorescence labeling with a chicken anti-AMIGO-1 polyclonal antibody in mouse neocortex (Peltola et al., 2011) that was distinct from that shown in other published studies (Kuja-Panula et al., 2003; Chen et al., 2012). To facilitate the characterization of the molecular properties and localization of AMIGO-1 in brain, we generated anti-AMIGO-1 rabbit polyclonal and mouse monoclonal antibodies in house. We validated the specificity of these antibodies for immunohistochemistry by comparing the signals obtained in samples prepared from wild-type (WT) and AMIGO-1 knockout (KO) mice. Figure 1 shows exemplar results of these validation experiments, which show images of somatosensory cortex from WT and KO mouse brain sections multiplex labeled with the anti-AMIGO-1 antibodies, and with the Hoechst 33258 nuclear dye, as an anatomical marker. Each of these newly generated anti-AMIGO-1 antibodies shows robust immunolabeling of neurons throughout neocortex in the samples prepared from WT mice (Figure 1). However, the strong AMIGO-1 immunoreactivity seen in WT samples is absent in the AMIGO-1 KO samples (Figure 1). These results suggest that the immunolabeling with these newly generated AMIGO-1 antibodies in immunohistochemistry analyses on mouse brain sections is specific for AMIGO-1, and that AMIGO-1 is primarily present on the cell bodies and proximal dendrites of neurons.

### AMIGO-1 is coexpressed and colocalized with Kv2.1 and Kv2.2 in diverse neuronal populations throughout mouse brain, and in diverse mammalian species

A previous study showed extensive overlap of immunofluorescence signals for AMIGO-1 and Kv2.1 in mouse neocortex (Peltola et al., 2011). We previously found extensive overlap of Kv2.1 and AMIGO-1 immunolabeling in striatal medium spiny neurons (Mandikian et al., 2014). Kv2.1 and Kv2.2 exhibit cell-type specific differences in expression throughout brain, particularly in neocortical (Kihira et al., 2010; Bishop et al., 2015) and basal forebrain (Hermanstyne et al., 2013) neurons. Whether AMIGO-1 is preferentially colocalized with Kv2.1 or Kv2.2 throughout the brain has not been determined.

**Figure 2:**
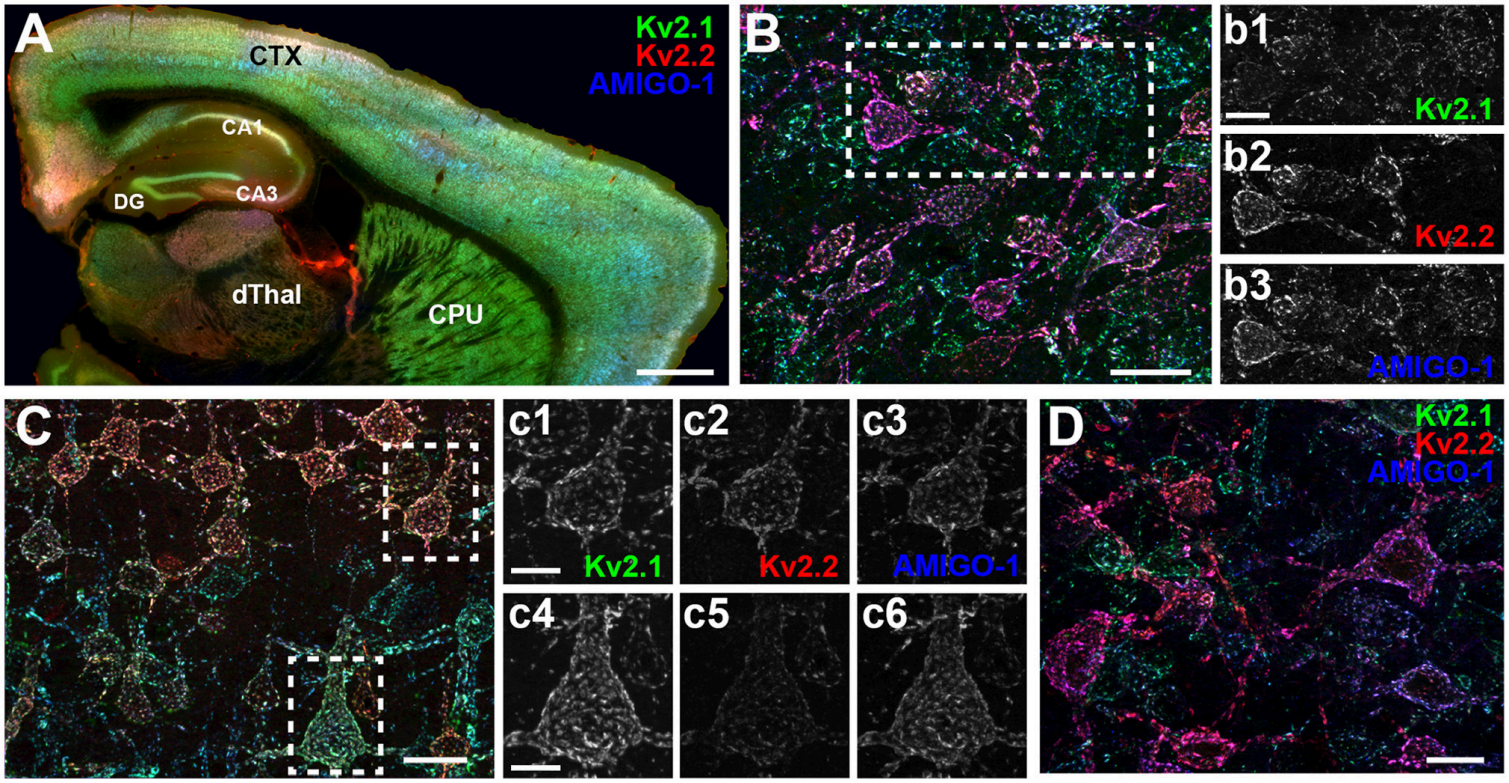
AMIGO-1 expression is highly colocalized with both Kv2.1 and Kv2.2 in mouse brain. **(A)** Sagittal section of mouse brain immunolabeled for Kv2.1 (green, K89/34 mAb), Kv2.2 (red, N372B/60 mAb), and AMIGO-1 (blue, L86/33 mAb). Caudate nucleus and putamen, CPU, Neocortex, CTX, *Cornu Ammonis* areas, CA1-3, dentate gyrus, DG, dorsal thalamus, dThal. Scale bar = 750 μm. **(B)** Immunolabeling for Kv2.1 (green), Kv2.2 (red), and AMIGO-1 (blue) in a sagittal section of mouse brain showing strong immunoreactivity of Kv2.2 and AMIGO-1 in cells of the magnocellular preoptic area (MCPO) of the hypothalamus. Scale bar = 20 μm. Separate grayscale images of individual layers for the boxed region are shown to the right. **(C)** Immunolabeling for Kv2.1 (green), Kv2.2 (red), and AMIGO-1 (blue) in a sagittal section of mouse brain showing strong immunoreactivity for AMIGO-1 in layer 5 somatosensory cortical neurons that differentially express Kv2.1 and Kv2.2. Scale bar = 20 μm. Separate grayscale images of individual layers are shown to the right for cells in layer 5a **(c1,c2,c3)** and layer 5b **(c4,c5,c6)**. Scale bar of insets = 10 μm. **(D)** Immunolabeling for Kv2.1 (green), Kv2.2 (red), and AMIGO-1 (blue) in a sagittal section of mouse brain showing strong immunoreactivity for AMIGO-1 among cells of the basal forebrain that differentially express Kv2.1 and Kv2.2. Scale bar = 20 μm. Note that areas of overlap between the three fluorescent signals appear white.

**Figure 3:**
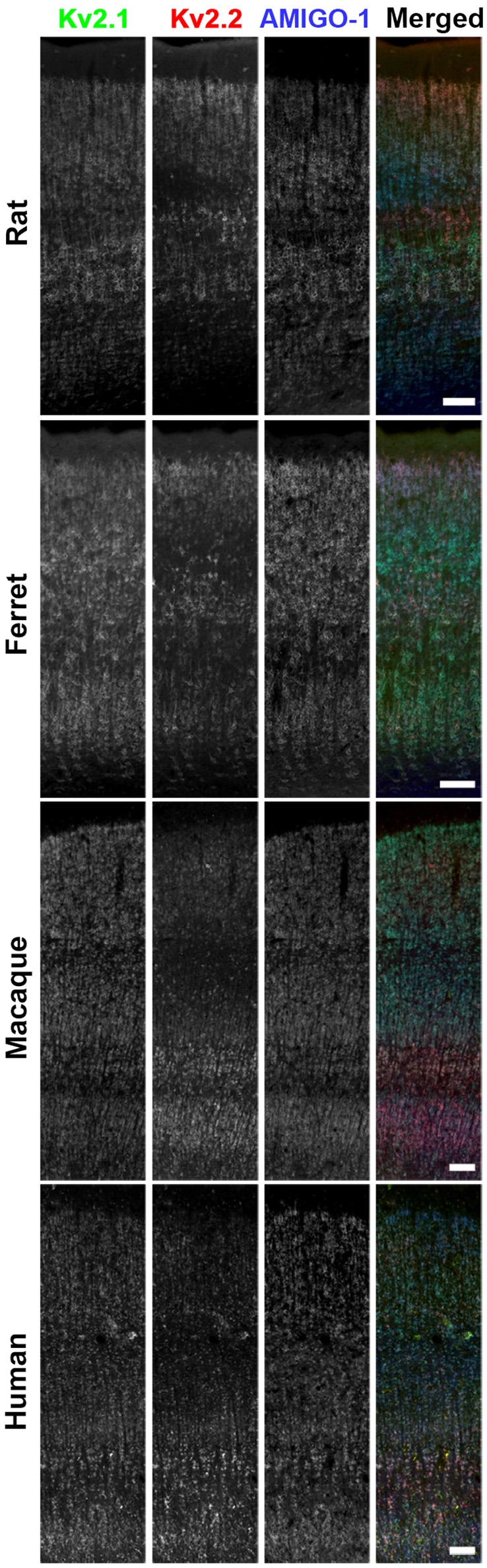
AMIGO-1 is coexpressed with Kv2 α subunits in multiple species. Brain sections from rat, ferret, macaque, and human immunolabeled for Kv2.1 (green, K89/34R mAb), Kv2.2 (red, N372B/1 mAb), and AMIGO-1 (blue, L86A/37 mAb). Images were adjusted linearly for optimal display. Scale bars = 100 μm.

We found that in mouse brain, AMIGO-1 immunolabeling exhibits extensive overlap with that of both Kv2.1 and Kv2.2 (Figure 2). This is evident in a whole brain sagittal section (Figure 2A), but also when examining specific brain regions and neuron classes that express high levels of immunoreactivity for either Kv2.1, Kv2.2, or for both Kv2 α subunits. For example, as shown in Figure 2B, in the ventromedial posterior nucleus of the thalamus (VMPO), Kv2.2 immunolabeling (red) is substantially stronger than that for Kv2.1 (green) in many neurons. AMIGO-1 immunolabeling (blue) is strong in neurons with high levels of Kv2.2, yielding a magenta signal in the merged image suggesting extensive overlap of red Kv2.2 and blue AMIGO-1 signals. However, other neurons that have Kv2.1 and AMIGO-1 immunolabeling exhibit a cyan signal (indicating overlap of green Kv2.1 and blue AMIGO-1). In neocortex (Figure 2C), neurons in layer 5A (Figures 2c1–c3), have prominent immunolabeling for Kv2.1, Kv2.2, and AMIGO-1, while those in layer 5b (Figures 2c4–c6) have predominantly Kv2.1 together with AMIGO-1. Lastly, different populations of neurons in basal forebrain (Figure 2D) exhibit prominent immunolabeling for either Kv2.1 or Kv2.2; however, both classes of neurons exhibit prominent AMIGO-1 labeling coclustered with either Kv2.1 or Kv2.2, with little apparent immunolabeling signal for AMIGO-1 alone (i.e., pure blue signal in Figure 2). Therefore, regardless of the levels of clustered Kv2.1 and Kv2.2 in a particular neuron type, AMIGO-1 is always found extensively coclustered with one or the other Kv2 α subunit, with no detectable pools of AMIGO-1 distinct from those present at the Kv2.1 or Kv2.2 clusters. Immunolabeling performed across neocortical samples from several species, including rat, ferret, macaque, and human, revealed that AMIGO-1 immunoreactivity that colocalizes with one or the other Kv2 α subunit is present in all of these species (Figure 3). Together, these results demonstrate strong colocalization of AMIGO-1 with Kv2.1 and/or Kv2.2, and that this pattern holds across multiple mammalian species.

### AMIGO-1 is precisely coclustered with both Kv2 α subunits

**Figure 4:**
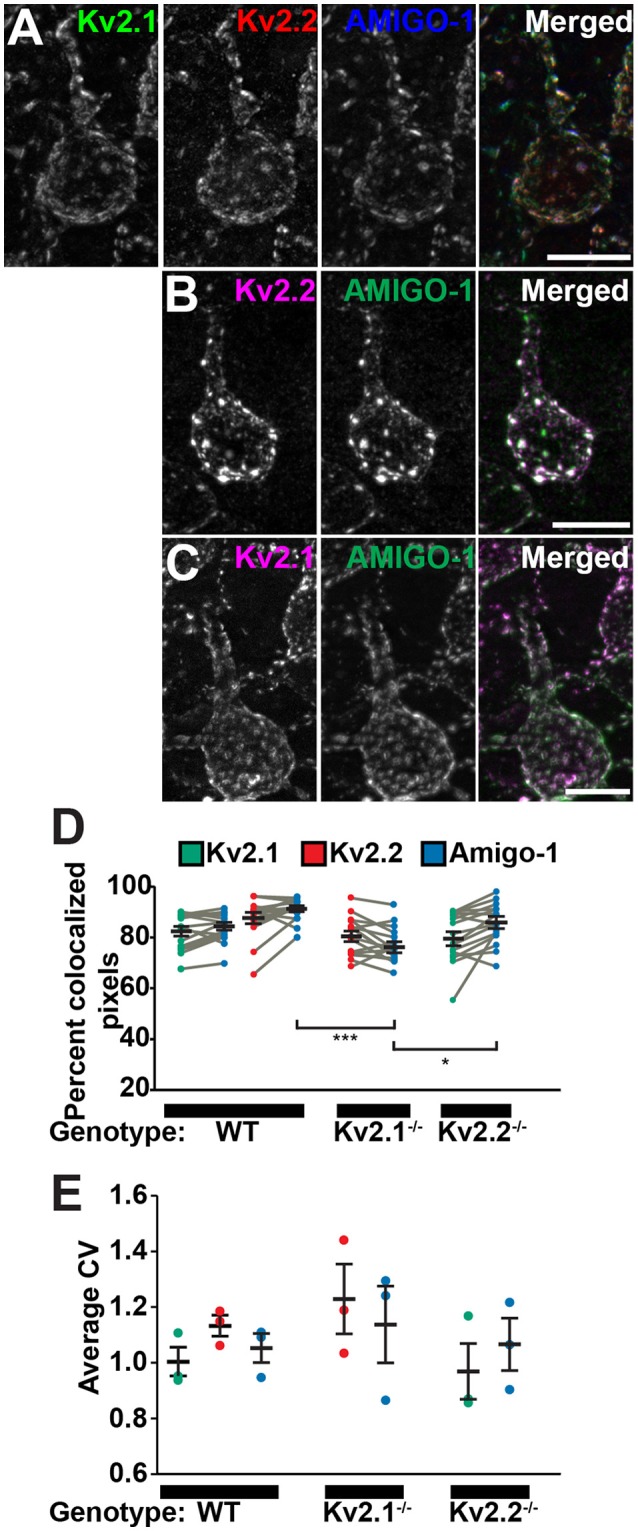
AMIGO-1 coclusters with Kv2.1 and Kv2.2 in neurons. High-magnification images of layer 5 pyramidal cells from S1 cortex of **(A)** WT mice immunolabeled for Kv2.1 (green, K89/34 mAb), Kv2.2 (red, N372B/60 mAb), and AMIGO-1 (blue, L86/33 mAb), and **(B)** Kv2.1 KO, and **(C)** Kv2.2 KO mice immunolabeled for Kv2 (magenta) and AMIGO-1 (green). Images were adjusted linearly for optimal display. Note that areas of overlap between the three fluorescent signals in **(A)**, and the two fluorescent signals in **(B,C)**, appear white. Scale bars = 10 μm. **(D)** Summary graph of MCC values measured for Kv2.1 (green) and Kv2.2 (red) and AMIGO-1 (blue) in cortical cells from WT, Kv2.1-KO and Kv2.2-KO animals (*n* = 16 cells each). Gray bars connect MCC values for Kv2 and AMIGO-1 from the same neuron. Error bars denote mean and SEM for each group. Differences in MCC values were evaluated by one-way ANOVA followed by Sidak's multiple-comparison test. ^***^p = 3.38 × 10^−6^, ^*^p = 0.0102. **(E)** Summary graph of CV measurements of cortical cells from WT, Kv2.1-KO and Kv2.2-KO mice immunolabeled for Kv2.1, Kv2.2 and AMIGO-1. Error bars represent the SEM for *n* = 3 groups. Differences in CV measurements between immunosignals were evaluated by one-way randomized block ANOVA followed by Sidak's multiple-comparison test. The p values for these pairwise comparisons of CV values were: Kv2.1 vs. Kv2.2 in WT: *p* = 0.554, Kv2.1 vs. AMIGO-1 in WT: *p* = 0.9605, Kv2.2 vs. AMIGO-1 in WT: *p* = 0.218, Kv2.2 vs. AMIGO-1 in Kv2.1^−/−^: *p* = 0.8672, Kv2.1 vs. AMIGO-1 in Kv2.2^−/−^: *p* = 0.9785. Note that the y-axis origin begins at 0.6.

Both Kv2.1 and Kv2.2 are present in brain neurons in large PM clusters (Trimmer, 1991; Scannevin et al., 1996; Kihira et al., 2010; Bishop et al., 2015) that are found over neuronal hypolemmal cisternae/ER:PM junctions (Du et al., 1998; Antonucci et al., 2001; Mandikian et al., 2014; Bishop et al., 2015; Fox et al., 2015) and apposed to astrocyte processes (Du et al., 1998). To more precisely determine the relationship of AMIGO-1 to these unique Kv2 clusters at the subcellular level, high-resolution immunofluorescence images were obtained from multiplex-labeled mouse brain sections. As shown in Figures 4A–C, high-magnification images of mouse cortical neurons demonstrate that AMIGO-1 precisely colocalizes with Kv2.1 and Kv2.2, whether the two Kv2 α subunits are coclustered with one another, or whether they are present in clusters comprised of primarily one or the other Kv2 α subunit. Analyses of colocalization using Manders' colocalization coefficient (MCC), which measures the co-occurrence of two signals regardless of their respective intensities (McDonald and Dunn, 2013) revealed extensive colocalization of AMIGO-1 with both Kv2.1 and Kv2.2 (Figure 4D). In cells from WT animals, on average 84% and 91% of AMIGO-1 immunosignal colocalized with Kv2.1 and Kv2.2, respectively. Similarly, in cells from Kv2.1 KO animals, an average of 77% of AMIGO-1 immunosignal co-occurred with Kv2.2, while in Kv2.2 KO animals an average of 85% of AMIGO-1 colocalized with Kv2.1 immunosignal. Interestingly, significantly more AMIGO-1 was found to colocalize with Kv2.1 in the Kv2.2 KO than with Kv2.2 in the Kv2.1 KO (AMIGO-1 in Kv2.2 KO, 85% vs. AMIGO-1 in Kv2.1 KO, 77%; *p* < 0.05). Additionally, significantly more AMIGO-1 was found to colocalize with Kv2.2 in cells from WT animals than in cells from Kv2.1 KO animals (AMIGO-1 colocalization with Kv2.2 in WT, 91% vs. AMIGO-1 colocalization with Kv2.2 in Kv2.1 KO, 77%; *p* < 0.001). Analyses of clustering using coefficient of variation of pixel intensity, a measure of variability and non-uniformity of fluorescence signals (Bishop et al., 2015), reveal that the populations of Kv2.1, Kv2.2, and AMIGO-1 in cortical neurons are present in clusters with similar characteristics (Figure 4E). These results show that in adult brain, AMIGO-1 is extensively associated with Kv2 α subunits, and that no autonomous AMIGO-1 populations with localizations distinct from that of Kv2.1 or Kv2.2 are apparent.

### AMIGO-1 is localized over hypolemmal subsurface cisternae at the ultrastructural level

**Figure 5:**
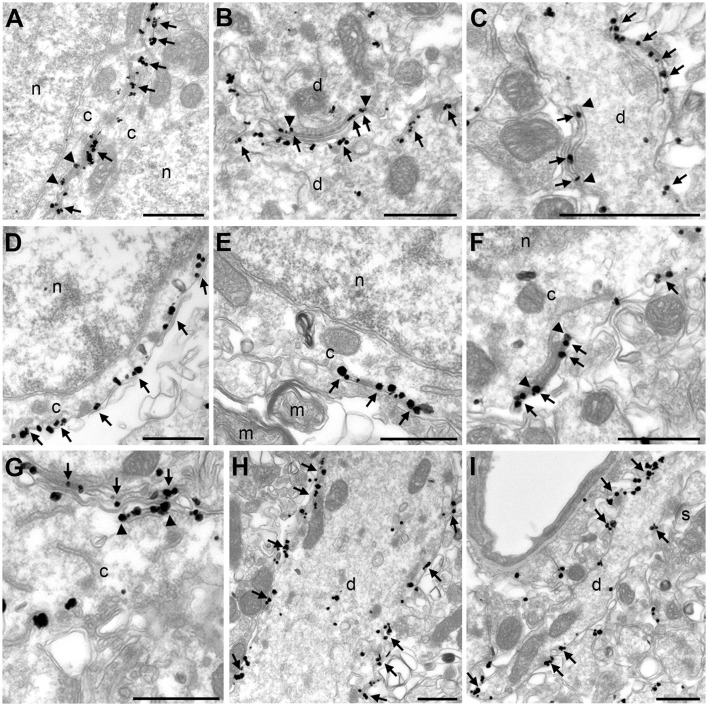
Kv2.1 and AMIGO-1 exhibit similar localization pattern at the ultrastructural level. Electron micrographs show the clustered localization of Kv2.1 immunogold particles (arrows) in the soma **(A)** and dendrite **(B,C)**. A vast majority of immunogold particles are PM associated. Similar to the localization of Kv2.1 shown here, and previous reports on Kv2.1 localization in rat (Du et al., 1998) and mouse (Mandikian et al., 2014), and Kv2.2 localization in mouse (Bishop et al., 2015), AMIGO-1 immunogold particles also show distinct clustering at the PM of the somata **(D–G)** and dendrites **(H)**. Immunogold particles are observed both in the large-caliber apical dendritic shaft **(H)** and the small-caliber oblique apical dendrites **(I)**. Noteworthy, both Kv2.1 and AMIGO-1 immunogold particles locate to the edges of subsurface cisternae (arrowheads in **A–C** for Kv2.1 and **F–G** for AMIGO). Note the lack of AMIGO-1 immunogold particles in myelinated axon and dendritic spines. *c*, cytoplasm; *d*, dendritic shaft; *m*, myelinated axon, *n*, nucleus, *s*, dendritic spine. Scale bars: 500 nm.

We used pre-embedding electron microscopic labeling in hippocampal CA1 pyramidal neurons to define the localization of Kv2.1 and AMIGO-1 at the ultrastructural level. As expected for subunits of a membrane protein, immunogold particles for Kv2.1 and AMIGO-1 were predominantly PM bound, and the rarely encountered cytosolic particles were generally associated with internal membranes. In good agreement with the cytoplasmic location of the epitopes of the mAbs used here, PM bound immunogold particles for Kv2.1 and AMIGO-1 were always observed in the vicinity of the inner leaflet of the PM. Interestingly, the ultrastructural localization pattern of AMIGO-1 exhibited striking similarities to the previously reported EM localization pattern of Kv2.1 (Du et al., 1998; Mandikian et al., 2014) and Kv2.2 (Bishop et al., 2015). In particular, both Kv2.1 and AMIGO-1 immunogold particles had a characteristic clustered pattern in the somatodendritic compartment (Figures 5A–I) and were located at the PM at sites corresponding to the edges of the juxtaposed hypolemmal subsurface cisternae (i.e., at ER:PM junctions) (Figures 5A–C,E–G). The labeling was also observed both in large (Figure 5H) and small caliber (Figure 5I) dendrites, presumably representing the apical dendritic shaft and oblique branches, respectively. Immunogold particles for Kv2.1 and AMIGO-1 were rarely observed in dendritic spines (data not shown) and were not detected in myelinated axons (Figure 5E) and presynaptic boutons. These results show that in adult brain neurons, AMIGO-1 is predominantly/exclusively present at PM sites typical of those containing Kv2 α subunits.

### Kv2.1 and Kv2.2 promote clustering of cell surface AMIGO-1 at ER:PM junctions in heterologous cells

**Figure 6:**
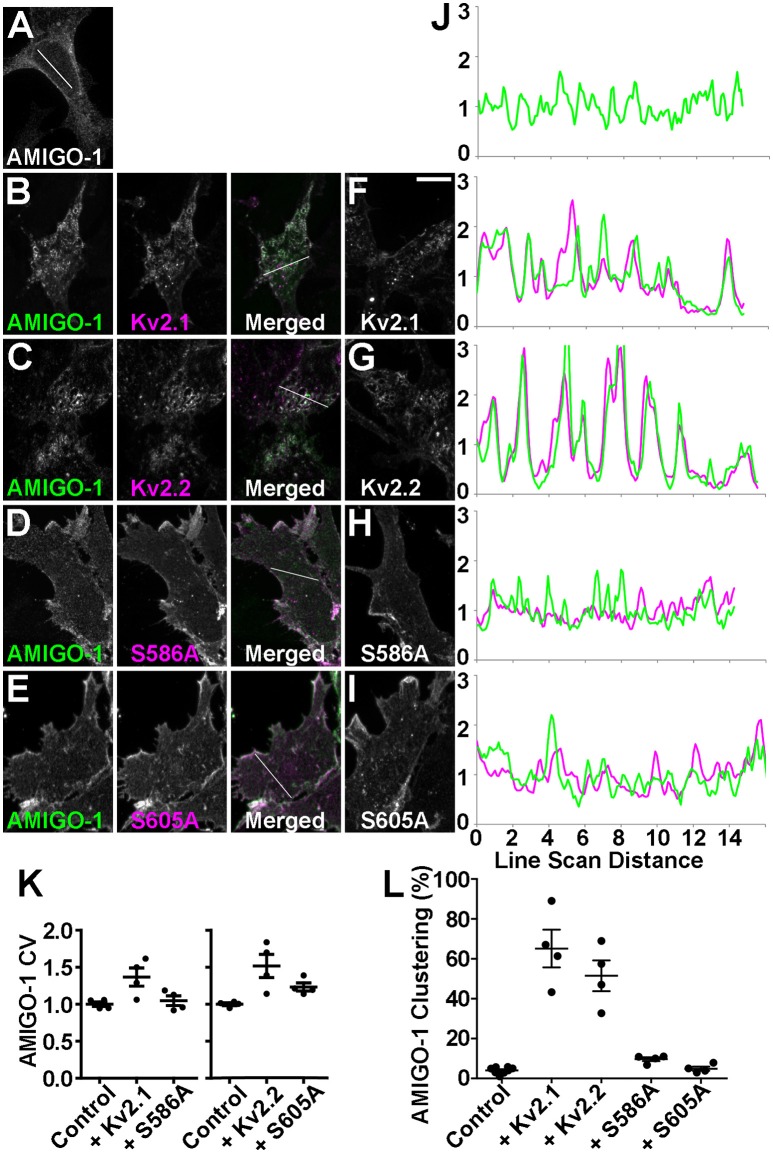
Kv2 α subunits promote clustering of AMIGO-1. HEK293 cells expressing **(A)** AMIGO-1 (green) alone, **(B)** AMIGO-1 (green) and Kv2.1 (magenta), **(C)** AMIGO-1 (green) and Kv2.2 (magenta), **(D)** AMIGO-1 (green) and S586A (magenta), and **(E)** AMIGO-1 (green) and S605A (magenta). Images were adjusted linearly for optimal display. Scale bar = 10 μm. HEK293 cells expressing **(F)** Kv2.1, **(G)** Kv2.2, **(H)** S586A, and **(I)** S605A alone. **(J)** AMIGO-1 (green) and Kv2 (magenta) fluorescence intensity values across individual line scans depicted by the white lines in the AMIGO-1 image in **(A)**, and the merged images in **(B–E)**. Note that areas of overlap between the two fluorescent signals appear white. **(K)** Coefficient of variation (CV) of AMIGO-1 fluorescence intensity in HEK293 cells expressing AMIGO-1 with the indicated Kv2 α subunit, relative to AMIGO-1 alone. Values are normalized to control. Data are the mean ± SEM from *n* = 4 independent samples of at least 14 cells each. The p values for these pairwise comparisons of CV values vs. cells expressing AMIGO-1 alone are: AMIGO-1 + Kv2.1: 0.0270, AMIGO-1 + S586A: 0.5366, AMIGO-1 + Kv2.2: 0.0167, AMIGO-1 + S605A: 0.0066. **(L)** HEK293 cells coexpressing AMIGO-1 with the indicated Kv2 α subunit were scored as having either clustered or unclustered AMIGO-1. Cells with over 25% of their membrane covered in clusters were considered clustered. Data are from an *n* = 4 independent samples of at least 100 cells each. The p values for these pairwise comparisons of clustering vs. cells expressing AMIGO-1 alone are: AMIGO-1 + Kv2.1: 2.345 × 10^−6^, AMIGO-1 + Kv2.2: 3.909 × 10^−6^, AMIGO-1 + S586A: 0.0004, AMIGO-1 + S605A: 0.4584. For other pairwise comparisons of clustering: AMIGO-1 + Kv2.1 vs. AMIGO-1 + S586A: *p* = 0.0011, AMIGO-1 + Kv2.2 vs. AMIGO-1 + S605A: *p* = 0.0010.

The lack of autonomous AMIGO-1 populations in mammalian brain neurons suggests Kv2 α subunits could play a role in organizing the subcellular localization of AMIGO-1. The initial report of AMIGO-1:Kv2.1 association showed that when coexpressed in heterologous HEK293 cells, AMIGO-1, and Kv2.1 exhibit extensive overlap in PM-associated clusters (Peltola et al., 2011) that are typical for Kv2.1 expressed in this cell background (Mohapatra and Trimmer, 2006; Kihira et al., 2010; Bishop et al., 2015). Recent studies have revealed that Kv2.2 (i.e., Kv2.2L; Kihira et al., 2010) is also clustered when expressed in HEK293 cells (Kihira et al., 2010; Bishop et al., 2015). Given the extensive coclustering of AMIGO-1 and Kv2.2 in certain brain neurons (Figures 2–4) we next investigated whether AMIGO-1 colocalizes with Kv2.2 when coexpressed in heterologous cells. AMIGO-1 was not clustered when expressed alone, and had a relatively uniform distribution (Figure 6A). However, we found that in HEK293 cells coexpressing AMIGO-1 with either Kv2.1 or Kv2.2, AMIGO-1 was present in large clusters that exhibited extensive overlap with the clustered immunofluorescence signals for these Kv2 α subunits (Figures 6B–C). Consistent with this, line scans across HEK293 cells reveal that the fluorescence intensities for AMIGO-1 (green) exhibit substantial variability, typical of non-uniform (clustered) localization, in cells coexpressing Kv2 α subunits (Figure 6J). The AMIGO-1 signals precisely overlap with those for Kv2 α subunits (magenta) in cells in which AMIGO-1 is coexpressed with either Kv2.1 or Kv2.2 (Figure 6J). Analysis of the AMIGO-1 immunofluorescence intensity across many HEK293 cells revealed that the CV of AMIGO-1 labeling intensity is significantly increased in the presence of WT Kv2.1 or Kv2.2 (Figure 6K), consistent with Kv2-induced AMIGO-1 clustering. A significantly increased incidence of cells with clustered AMIGO-1 was also observed upon coexpression with either Kv2.1 or Kv2.2 (Figure 6L). Together these data suggest that association with a clustered Kv2 α subunit induces clustering of otherwise uniformly expressed AMIGO-1.

To further determine whether these Kv2 α subunits are the primary determinants of AMIGO-1 clustering, we coexpressed AMIGO-1 with Kv2 α subunits carrying single Serine to Alanine point mutations in their clustering domains that eliminate clustering and yield uniform PM localization (Lim et al., 2000; Bishop et al., 2015). We found that coexpression of AMIGO-1 with either the Kv2.1 S586A (Lim et al., 2000) or the Kv2.2 S605A (Bishop et al., 2015) clustering mutants failed to induce clustering of AMIGO-1 as seen upon coexpression with the WT Kv2 α subunits (Figures 6D,E,J). The CV of AMIGO-1 labeling intensity is also not altered in the presence of the non-clustering Kv2.1 S586A mutant (Figure 6K), although coexpression of AMIGO-1 with the non-clustering Kv2.2 S605A mutant did yield a small but significant increase in the CV of AMIGO-1 labeling intensity, even though distinct AMIGO-1 clusters were not apparent in these cells (Figure 6K). Consistent with these overall results, coexpression of AMIGO-1 with the non-clustering Kv2 mutants lead to a reduction in the incidence of cells with clustered AMIGO-1 compared to WT (Figure 6L). We found that although AMIGO-1 clustering was induced by coexpression with either Kv2.1 or Kv2.2, coexpression with AMIGO-1 did not affect the subcellular localization of WT or mutant Kv2.1 or Kv2.2 (Figures 6F–I). These results together suggest that AMIGO-1 is not competent to form clusters on its own, that Kv2 α subunits are the primary determinants of AMIGO-1 clustering, and that AMIGO-1 coexpression does not significantly impact the clustering of coexpressed Kv2 α subunits.

**Figure 7:**
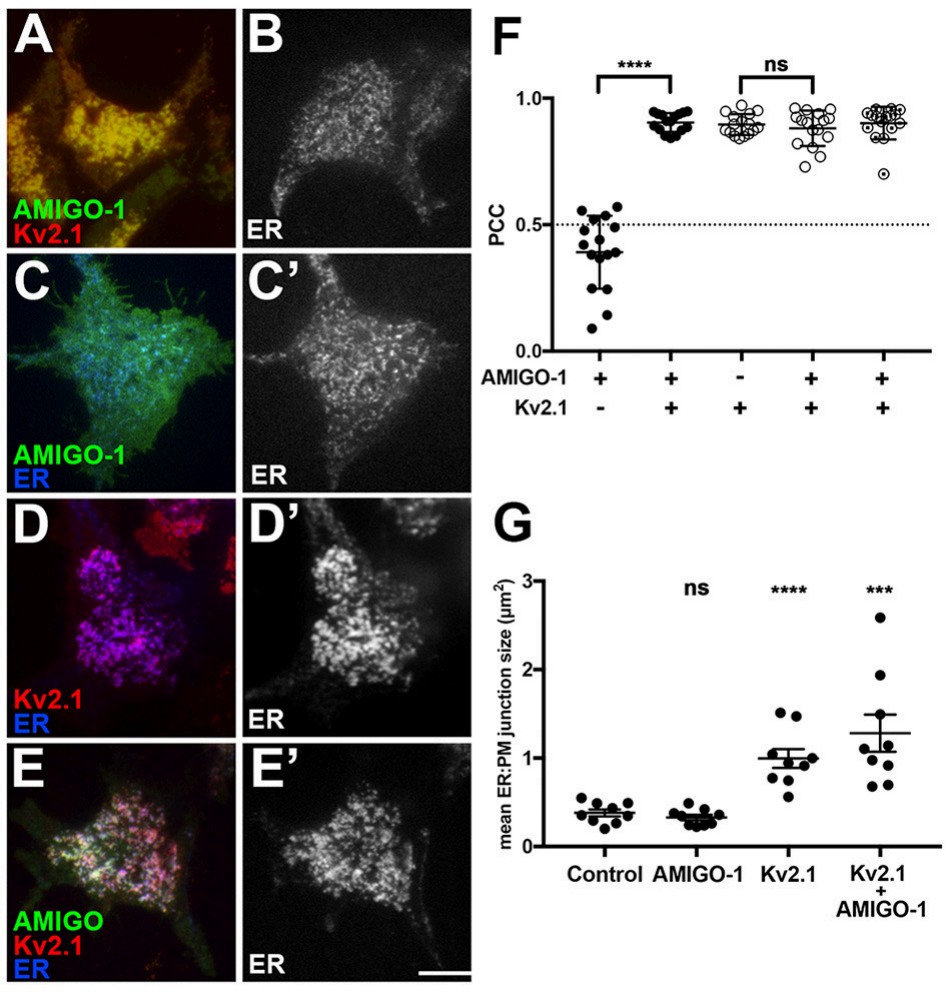
Kv2.1:AMIGO-1 complexes are present at and recruit/stabilize ER:PM junctions. Live cell TIRF imaging. **(A)** Cell expressing YFP-AMIGO-1 (green) and DsRed-Kv2.1 (red). **(B)** Untransfected cell showing ER (SEC61β) labeling. **(C)** Cell expressing YFP-AMIGO-1 (green) showing ER (SEC61β, blue) labeling. **(C**′**)** ER (SEC61β) labeling of cell in **(C)**. **(D):** Cell expressing DsRed-Kv2.1 (red) showing ER (SEC61β, blue) labeling. **(D**′**)**: ER (SEC61β) labeling of cell in **(D)**. **(E)** Cell expressing YFP-AMIGO-1 (green) and DsRed-Kv2.1 (red) showing ER (SEC61β, blue) labeling. **(E**′**)** ER (SEC61β) labeling of cell in panel E. Note that areas of overlap between the three fluorescent signals appear white. Scale bar = 10 μm. **(F)** Graph of Pearson's Colocalization Coefficient (PCC) values for SEC61β and AMIGO-1 (filled circles), or SEC61β and Kv2.1 (open circles). Partially filled circles are PCC values for AMIGO-1 and Kv2.1. Bars are the mean ± SEM. ^****^p = 1.62 × 10^−14^. ns: *p* = 0.4798. **(G)** Mean ER:PM junction size (in μm^2^) for cells expressing SEC61β ER marker alone, or with AMIGO-1 and/or Kv2.1 as indicated. Bars are the mean ± SEM. ns: *p* = 0.3119. ^****^p = 5.275 × 10^−5^. ^***^p = 6.463 × 10^−4^.

In brain neurons, Kv2.1 and AMIGO-1 are localized in the PM over hypolemmal subsurface cisternae (Figure 5). In certain neurons Kv2.1 is found localized over clustered ryanodine receptors (Antonucci et al., 2001; Mandikian et al., 2014), and overexpression of Kv2.1 in cultured hippocampal neurons enhances clustering of ryanodine receptors at sites of Kv2.1 clustering (Antonucci et al., 2001). Recent studies revealed that exogenous Kv2.1 expression can recruit/stabilize ER at the PM, and enhance ER:PM junctions, in heterologous cells and neurons (Cobb et al., 2015; Fox et al., 2015). Given the apparent presence of AMIGO-1 in many native Kv2.1 complexes, we next addressed whether Kv2.1:AMIGO-1 complexes could recruit/stabilize ER as occurs upon exogenous expression of Kv2.1 alone (Cobb et al., 2015; Fox et al., 2015). We coexpressed DsRed-tagged Kv2.1, YFP-tagged AMIGO-1 and BFP-tagged SEC61β (a general ER marker) in HEK293 cells and performed live cell TIRF imaging to selectively visualize PM and near PM ER (i.e., ER:PM junctions). We found that similar to the results obtained using immunocytochemistry on fixed cells (Figure 6), in live cells the cell surface Kv2.1 and AMIGO-1 precisely colocalized with one another (Figure 7A), as shown by their high PCC values (0.90 ± 0.065, *n* = 16 cells; Figure 7F). The uniformly expressed PM AMIGO-1 signal in cells expressing AMIGO-1 alone (Figure 7C) was not colocalized with the reticular/tubular ER signal (Figure 7C′) (PCC values 0.39 ± 0.14, *n* = 16 cells). However, when coexpressed with Kv2.1, AMIGO-1 exhibited prominent colocalization with the resultant ER:PM junctions (Figure 7E), with PCC values (0.90 ± 0.037, *n* = 16 cells) significantly higher (*p* = 1.62 × 10^−14^) than in cells expressing AMIGO-1 alone (Figure 7F). Kv2.1 expressed alone colocalized with associated ER:PM junctions (Figure 7D), and had PCC values (0.90 ± 0.041, *n* = 16 cells; Figure 7F) not significantly different (*p* = 0.4798) than those in cells coexpressing Kv2.1 and AMIGO-1 (Figure 7E) (0.88 ± 0.070, *n* = 16 cells; Figure 7F). Moreover, the mean size of ER:PM junctions on a per cell basis were significantly increased (Figure 7G) by expression of Kv2.1 alone (Figure 7D′; mean ER:PM junction size 1.00 ± 0.32 μm^2^, *n* = 9 cells, *p* = 5.275 × 10^−5^ vs. untransfected cells), and by coexpression of Kv2.1 and AMIGO-1 (Figure 7E′; mean ER:PM junction size 1.28 ± 0.60 μm^2^, *n* = 9 cells, *p* = 6.463 × 10^−4^ vs. untransfected cells), but not by expression of AMIGO-1 alone (Figure 7C′; mean ER:PM junction size 0.33 ± 0.092 μm^2^, *n* = 9 cells, *p* = 0.3119 vs. untransfected cells, mean ER:PM junction size 0.38 ± 0.12 μm^2^, *n* = 9 cells). An untransfected cell example is shown in Figure 7B. These results show that when expressed alone in heterologous cells, AMIGO-1 is not present at ER:PM junctions, but when coexpressed with Kv2.1 the Kv2.1:AMIGO-1 complexes exist at the enhanced ER:PM junctions present in cells expressing Kv2.1. It also illustrates that the Kv2.1:AMIGO-1 complexes can recruit/stabilize ER at these sites, but that AMIGO-1 can neither recruit/stabilize these junctions on its own, nor influence the ability of Kv2.1 to impact these junctions.

### AMIGO-1 localization in brain neurons is impacted by genetic ablation of Kv2 α subunit expression

**Figure 8:**
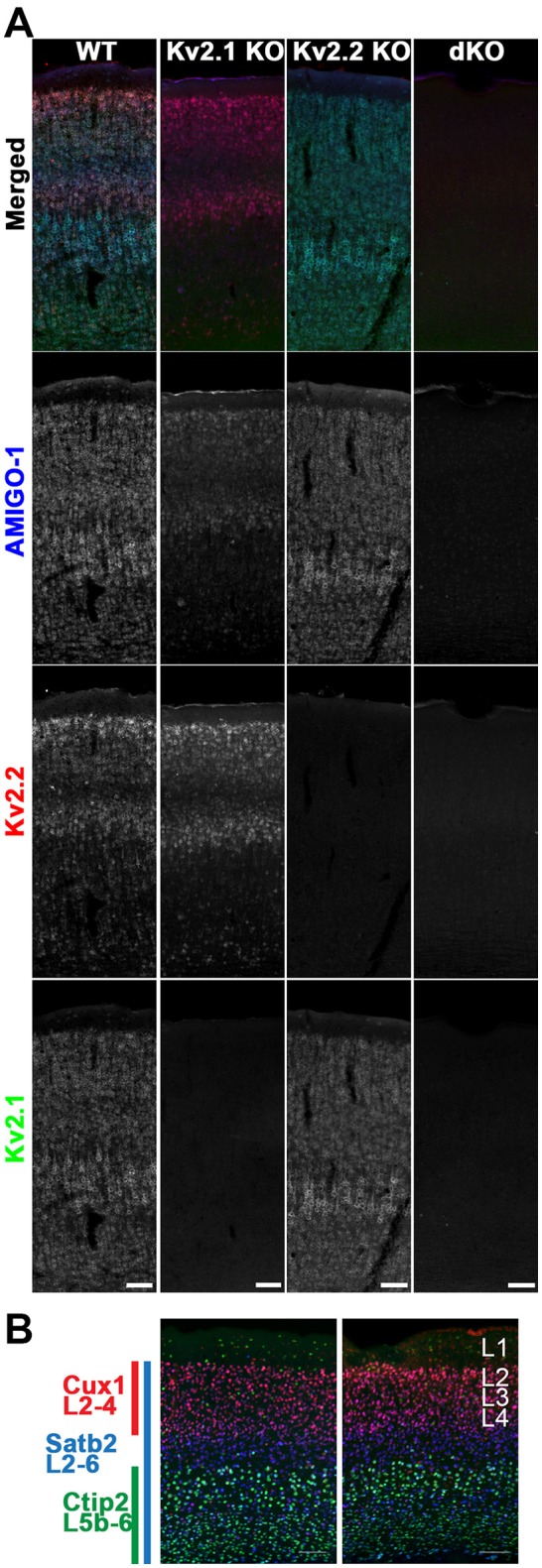
Mice lacking Kv2 α subunits have altered AMIGO-1 expression. **(A)** Mouse sagittal brain sections from WT, Kv2.1 KO, Kv2.2 KO, and Kv2 dKO mice immunolabeled for AMIGO-1 (blue, AMIGO-1 pAb), Kv2.2 (red, N372B/60 mAb), and Kv2.1 (green, K89/34 mAb). Note the lack of AMIGO-1 labeling present in the Kv2 double KO mice. Scale bar = 100 μm. **(B)** Sagittal brain sections from WT and Kv2 dKO mice immunolabeled for cortical layer markers Cux1 (red), Satb2 (blue), and Ctip2 (green). Cortices of dKO mice show no gross abnormalities in cortical laminae or cell density. Scale bar = 100 μm. Note that areas of overlap between the three fluorescent signals appear white.

Given the results above that demonstrate extensive coexpression and colocalization of AMIGO-1 with Kv2.1 and Kv2.2 in mammalian brain neurons, and the requirement for Kv2 α subunit coexpression for clustering of AMIGO-1 at ER:PM junctions in heterologous cells, we next determined how ablation of expression of Kv2 α subunits in KO mice would impact the clustered subcellular localization of AMIGO-1 at specific sites in brain neurons *in vivo*. Figure 8A shows labeling for Kv2.1 (green) and Kv2.2 (red), in conjunction with AMIGO-1 labeling (blue) in mouse brain sagittal sections prepared from WT, Kv2.1 KO and Kv2.2 KO mice. As is apparent in both the Kv2.1 KO and the Kv2.2 KO sections, while AMIGO-1 signal is still present, it is only retained in the neurons that express the remaining Kv2 α subunit, and is absent in neurons in which expression of the predominant Kv2 α subunit was eliminated in the KO. This is especially clear in neocortex, as unlike the widespread expression of Kv2.1, Kv2.2 expression is limited primarily to cortical layers 2 and 5a (Bishop et al., 2015). Following genetic ablation of Kv2.1 expression, AMIGO-1 labeling in neocortex is now restricted to these specific layers, and is lost in the other neocortical layers that would normally express high levels of Kv2.1 (Figure 8A).

The restricted cellular expression of AMIGO-1 in the Kv2.1 KO in only those neurons with robust Kv2.2 expression suggests that maintenance of AMIGO-1 may require expression of a Kv2 α subunit. To determine if this is the case, we generated homozygous Kv2.1/Kv2.2 double knockout mice (Kv2 dKO) by crossing Kv2.1 heterozygotes (Kv2.1^+/−^) to Kv2.2 homozygous KO mice (Kv2.2^−/−^). Importantly, the gross brain morphology is maintained in the Kv2 dKO (data not shown), and immunolabeling with the neocortical layer markers Cux1 (L2-4), Satb2 (L2-6), and Ctip2 (L5b-6) revealed no gross abnormalities in neocortical layering or cell density in the dKO mouse brain samples compared to those from WT mice (Figure 8B). However, no clear AMIGO-1 immunolabeling signal was apparent in the Kv2 dKO samples (Figure 8A). Similar results were obtained using multiple Abs against AMIGO-1 (data not shown), including two different mouse mAbs with distinct cytoplasmic domain binding sites (L86/33 and L86A/37), and a rabbit pAb (AMIGO-1) made against the intracellular C-terminus, as well as a mouse mAb (L98/12) directed against the extracellular N-terminus, suggesting that this is not likely due to a mere change in AMIGO-1 immunoreactivity, but to decreased AMIGO-1 expression. These results suggest that AMIGO-1 expression is substantially reduced in the absence of Kv2 α subunit expression in adult brain neurons *in vivo*.

### Kv2.1 and Kv2.2 promote AMIGO-1 cell surface expression in heterologous cells

**Figure 9:**
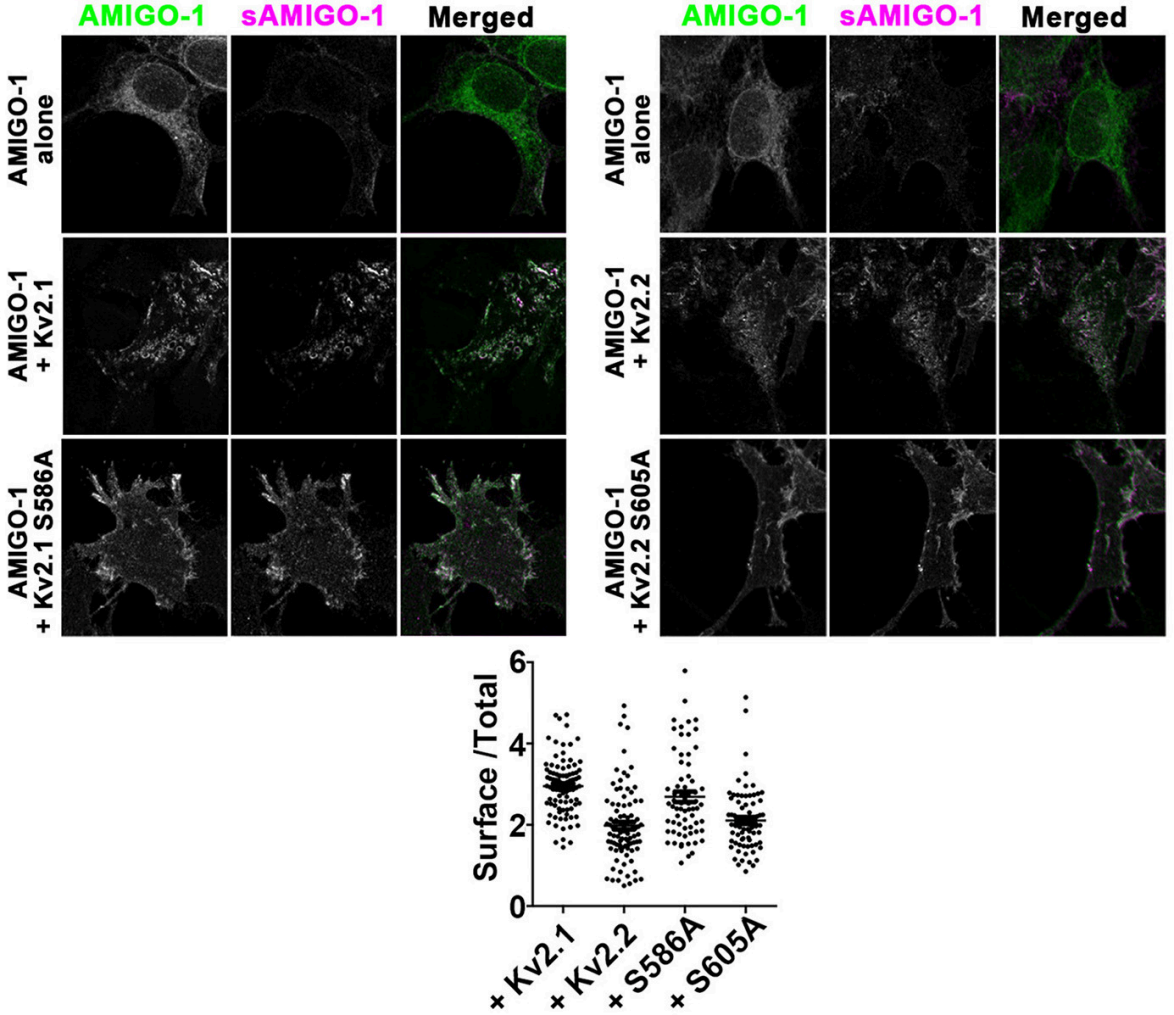
Kv2 α subunits increase AMIGO-1 cell surface localization in heterologous cells. HEK293 cells expressing AMIGO-1 alone, or AMIGO-1 plus WT Kv2.1 or Kv2.2, or the respective clustering deficient mutants Kv2.1 S586A or Kv2.2 S605A, as indicated. Cells were immunolabeled for total AMIGO-1 (green) and cell surface AMIGO-1 (magenta). All images within the left and right panels were acquired under identical conditions and were linearly adjusted identically. Note that areas of overlap between the two fluorescent signals appear white. Scale bar = 10 μm. The graph shows the ratio of cell surface: total AMIGO-1 fluorescence intensity measured in HEK293 cells expressing AMIGO-1 alone or with the indicated Kv2 α subunits. Values are normalized to cells expressing AMIGO- alone. Data are the mean ± SEM from 4 independent samples of at least *n* = 67 cells total. The p values (two-tailed unpaired *t*-test) for surface/total AMIGO-1 levels relative to AMIGO-1 alone: AMIGO-1 + Kv2.1: 2.192 × 10^−37^, AMIGO-1 + S586A: 4.681 × 10^−20^, AMIGO-1 + Kv2.2: 8.240 × 10^−13^, AMIGO-1 alone vs. AMIGO-1 + S605A: 9.921 × 10^−17^.

Given the substantial loss of AMIGO-1 immunolabeling in brain sections upon elimination of Kv2 α subunit expression *in vivo*, we next examined how Kv2 α subunits influence AMIGO-1 expression in heterologous cells. Immunolabeling of cell surface AMIGO-1 in intact cells expressing AMIGO-1 alone using a mAb (L98/12) targeting an extracellular region of AMIGO-1 revealed uniform diffuse PM-associated labeling (Figure 9). Immunolabeling of total AMIGO-1 in permeabilized cells revealed a substantial intracellular AMIGO-1 population (Figure 9). The level of cell surface AMIGO-1 increased, and the level of the intracellular AMIGO-1 decreased, upon coexpression with Kv2 α subunits, indicating a redistribution of the intracellular AMIGO-1 population to the cell surface (Figure 9). The enhancement of the cell surface AMIGO-1 population was observed upon coexpression of AMIGO-1 with both the clustered WT Kv2 α subunits, as well as with the non-clustered Kv2.1 S586A and Kv2.2 S605A point mutants (Figure 9). This indicates that the ability of Kv2 α subunits to enhance AMIGO-1 cell surface expression is independent of formation of PM clusters.

**Figure 10:**
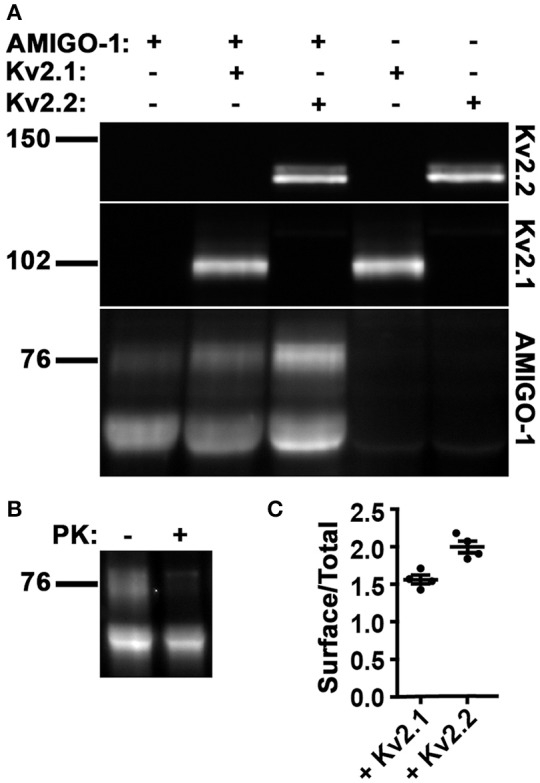
Kv2 α subunits increase cell surface expression of AMIGO-1. **(A)** Representative immunoblot of HEK293 cell lysates expressing AMIGO-1, AMIGO-1 + Kv2.1, AMIGO-1 + Kv2.2, Kv2.1, or Kv2.2. Numbers to the left of immunoblots indicate the mobility of molecular mass standards in kD. See Figure S1 for original immunoblot. **(B)** Representative immunoblot of AMIGO-1 expressing HEK293 cells treated with or without proteinase K (PK). Numbers to the left of the immunoblots indicate the mobility of molecular mass standards in kD. See Figure S2 for original immunoblot. **(C)** The ratio of the intensity of AMIGO-1 cell surface (top band) to total (sum of top and bottom bands) was measured in HEK293 cells expressing AMIGO-1 alone (control), AMIGO-1 with Kv2.1 (white bar), or AMIGO-1 with Kv2.2 (black bar). Values are normalized to control. Data are the mean ± SEM from *n* = 4 independent samples. The *p* (two-tailed unpaired *t*-test) for surface/total AMIGO-1 levels relative to AMIGO-1 alone: AMIGO-1 + Kv2.1: 0.0003, AMIGO-1 + Kv2.2: 2.6111 × 10^−5^.

We next used immunoblots to further characterize the cell surface and intracellular populations of AMIGO-1 expressed in heterologous cells, without and with Kv2 α subunit coexpression. Immunoblotting for AMIGO-1 reveals two populations with distinct electrophoretic mobility, one of M_r_ ≈ 76 kD, and one of M_r_ ≈ 66 kD (Figure 10A). These electrophoretically distinct AMIGO-1 populations arise from distinct posttranslational processing of N-linked sugar chains on the AMIGO-1 glycoprotein (Kajander et al., 2011), presumably reflecting distinct locations within the cell. To confirm this, we employed an assay in which live cells are treated with the membrane impermeant protease Proteinase K to selectively digest the extracellular domains of PM localized proteins (Zhou et al., 1998; Manganas and Trimmer, 2000). We found that the M_r_ ≈ 76 kD AMIGO-1 population was selectively eliminated by Proteinase K treatment, while the levels of the M_r_ ≈ 66 kD population remained unaffected (Figure 10B). This supports that the M_r_ ≈ 76 kD population corresponds to cell surface AMIGO-1, while the M_r_ ≈ 66 kD population corresponds to intracellular AMIGO-1. We next assessed whether Kv2 α subunit coexpression alters the cell surface expression levels of AMIGO-1 by immunoblotting samples from HEK293 cells expressing AMIGO-1 alone or coexpressing Kv2 α subunits. A significant increase in the cell surface (M_r_ ≈76 kD) AMIGO-1 population was seen upon coexpression of either Kv2.1 or Kv2.2 (Figure 10A). We also observed an increase in the ratio of the cell surface (M_r_ ≈76 kD) vs. total (M_r_ ≈ 76 kD + M_r_ ≈ 66 kD) AMIGO-1 populations (Figure 10C). This further demonstrates that coexpression with either Kv2.1 or Kv2.2 facilitates intracellular trafficking of AMIGO-1 and promotes a redistribution of intracellular AMIGO-1 to the cell surface.

### The expression level and posttranslational processing of AMIGO-1 in adult brain is impacted by loss of Kv2 α subunit expression

**Figure 11:**
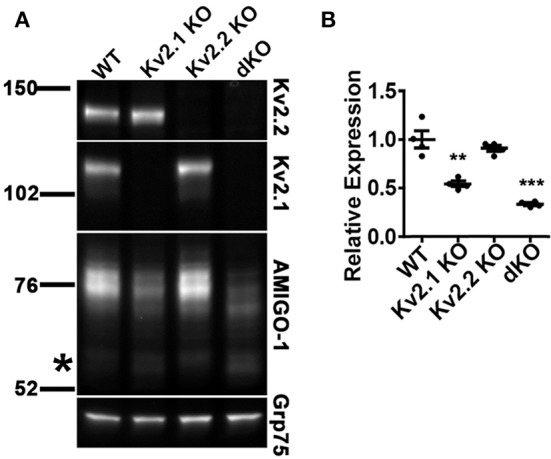
AMIGO-1 cell surface expression is preferentially decreased in mice lacking Kv2 α subunits. **(A)** Representative immunoblot of crude whole brain homogenates from WT, Kv2.1 KO, Kv2.2 KO, and Kv2 double KO mice. Immunoblots were probed with mAbs against Kv2.1 (K89/34 mAb), Kv2.2 (N372B/60 mAb), AMIGO-1 (AMIGO-1 pAb), and Grp75 (N52A/42 mAb) as a loading control. Note that AMIGO-1 signal in higher M_r_ bands is substantially reduced in both Kv2.1 KO and Kv2 dKO as compared to WT. Numbers to the left of the immunoblots indicate the mobility of molecular mass standards in kD. The asterisk to left of the AMIGO-1 panel shows approximate location AMIGO-1 population of M_r_ = 66 kD. See Figure S3 for original immunoblot. **(B)** Summary graph of differences in AMIGO-1 protein levels between WT and KO mice. Fluorescence intensity values were normalized to the loading control (Grp75) and then expressed relative to the total WT signal. Data are the mean ± SEM from *n* = 4 mice per group. Differences in AMIGO-1 expression between WT and Kv2.1 KO as well as WT and Kv2 dKO were statistically significant as evaluated by independent *t*-test (Kv2.1 KO, *p* = 0.002; Kv2.2 KO, *p* = 0.36; Kv2 dKO, *p* = 0.004).

Our immunohistochemical results suggest that the expression levels of AMIGO-1 are strikingly reduced in the brains of Kv2 dKO mice. We performed immunoblot analyses on WT, Kv2.1 KO, Kv2.2 KO, and Kv2 dKO whole brain homogenates, and confirmed that the individual Kv2 α subunits were absent in their respective KO samples, and in the Kv2 dKO samples. Largely consistent with the immunohistochemistry results above (Figure 8), the overall levels and characteristics of the population of AMIGO-1 detected with a mAb against the intracellular C-terminus were largely maintained in the Kv2.2 KO (Figures 11A,B). However, the overall levels of AMIGO-1 were significantly reduced in samples from the Kv2.1 KO mice, and from the Kv2 dKO mice (Figures 11A,B). Notably, in the samples from the Kv2 dKO mice, the levels of the M_r_ ≈ 76 kD cell surface population of AMIGO-1 were significantly reduced compared to samples from WT mice (Figure 11A). As such, a higher proportion of the AMIGO-1 signal in both the Kv2.1 KO and the Kv2 dKO homogenates was present in the lower M_r_ population than in the WT or Kv2.2 KO samples. These results suggest both an overall decrease in AMIGO-1 expression levels, as well as reduced PM trafficking, as evidenced by reduced levels of the higher M_r_ ≈76 kD cell surface population, and that the remaining AMIGO-1 is present in the lower M_r_ intracellular populations. Taken together, these results suggest that Kv2 expression is necessary for the proper expression and cell surface localization of AMIGO-1 in adult brain neurons *in vivo*.

## Discussion

Here we show that AMIGO-1 extensively colocalizes with both Kv2.1 and Kv2.2 in neurons throughout the brain in diverse mammalian species. In fact, there is no apparent AMIGO-1 population outside of that colocalized with Kv2 α subunits that would suggest a role for AMIGO-1 in adult brain outside of being an auxiliary subunit of Kv2 channels. Moreover, the presence of one or another Kv2 α subunit is needed to maintain overall expression and localization of AMIGO-1 in adult brain neurons.

The expression and localization of AMIGO-1 reported herein are generally consistent with more limited published immunohistochemical analyses of AMIGO-1, which demonstrated colocalization of AMIGO-1 and Kv2.1 in adult mouse neocortex (Peltola et al., 2011) and striatum (Mandikian et al., 2014). These studies, and the results presented here, suggest a cellular and subcellular localization of AMIGO-1 on the cell bodies and proximal dendrites of diverse populations of neurons throughout the brain. However, the overall pattern of AMIGO-1 localization shown here is distinct from that shown in the original study of AMIGO-1 expression and localization in rat (Kuja-Panula et al., 2003), and mouse (Chen et al., 2012) brain, which used anti-AMIGO-1 antibodies distinct from one another, and distinct from those used here. The basis for the distinct immunolabeling patterns obtained with these different anti-AMIGO-1 Abs is not known. The results presented here, obtained using a diverse panel of KO-validated monoclonal and polyclonal anti-AMIGO-1 Abs with distinct binding sites on AMIGO-1, in general yielded a pattern of localization consistent with that reported for mouse neocortex in a recent study (Peltola et al., 2011). We note that in addition to its presence on the soma and proximal dendrites of adult brain neurons, AMIGO-1 immunolabeling is also present on the axon initial segment (Bishop and Trimmer, unpublished data), the other major site of Kv2 channel expression (Johnston et al., 2008; Sarmiere et al., 2008; Sánchez-Ponce et al., 2012; King et al., 2014).

The clustered localization of AMIGO-1 on the soma and proximal dendrites of adult brain neurons is interesting in light of the role of AMIGO-1 during development as an adhesion molecule involved in development of neuronal circuitry. Knockdown of AMIGO-1 expression in developing zebrafish impairs the formation of fasciculated fiber tracts and disturbs the development of dopaminergic circuits (Zhao et al., 2014). However there are no gross anatomical abnormalities in the brains of constitutive global AMIGO-1 KO mice (Peltola et al., 2016), or, as shown here, in Kv2 dKO mice, which also have greatly reduced levels of AMIGO-1 expression, particularly in the neuronal PM. Future studies investigating the expression and localization of AMIGO-1 during development in WT and Kv2 KO mice may shed light on whether AMIGO-1 plays a distinct role as a cell adhesion protein promoting axon growth and fasciculation (Kuja-Panula et al., 2003) and/or dendritic outgrowth (Chen et al., 2012) during early mouse development, and how this role is impacted by Kv2 α subunits.

We found that expression and trafficking of AMIGO-1 in adult mouse brain and in heterologous cells is enhanced by Kv2 α subunits. It is interesting that manipulating the expression of one or the other Kv2 α subunit so profoundly alters the overall expression level and subcellular localization of AMIGO-1, yet has little impact on the expression of the remaining Kv2 α subunit paralog [also see (Speca et al., 2014; Bishop et al., 2015)]. That this phenomenon was observed using distinct immunohistochemical and biochemical assays employing multiple anti-AMIGO-1 and Kv2 Abs with distinct binding sites is supportive that these changes in AMIGO-1 immunolabeling reflect changes in AMIGO-1 protein expression itself, as opposed to changes in AMIGO-1 immunoreactivity. Based on our heterologous cell results, we speculate that inefficient trafficking and intracellular retention of AMIGO-1, leading to enhanced degradation, results in the reduced levels of AMIGO-1 seen in adult brain neurons lacking Kv2 α subunits. A similar loss of auxiliary subunit expression upon elimination of partner principal subunits was previously observed for the cytoplasmic KChIP auxiliary subunits of Kv4 channels (Menegola and Trimmer, 2006), whose stability is reduced in the absence of their partner Kv4 α subunit (Foeger et al., 2010). Whether Kv2 channel expression is similarly required to support the function of AMIGO-1 as homophilic cell adhesion molecule underlying neurite outgrowth and fasciculation during development (Kuja-Panula et al., 2003; Chen et al., 2012) is not known.

We note that the almost complete loss of AMIGO-1 immunolabeling signal in the Kv2 dKO brain sections in our immunohistochemistry experiments (Figure 8) is more substantial than that observed in our biochemical experiments (Figure 11). Our immunoblot data suggest that the bulk of the AMIGO-1 retained intracellularly in dKO neurons is within (and likely throughout) the ER, as modifications to the N-linked glycans on AMIGO-1 that typically occur in the Golgi apparatus and yield altered electrophoretic mobility are lacking in the samples from the dKO mice. It is plausible that the loss of AMIGO-1 immunolabeling intensity in brain sections is due to a dilution effect when comparing the highly clustered AMIGO-1 in the PM to that retained in and distributed throughout the ER. We calculate, using a sphere with diameter of 20 μm to approximate a neocortical neuron cell body, that intracellular/ER retention would result in an ≈60-fold reduction in maximum signal intensity relative to the same amount of protein concentrated in the PM (representing ≈0.15% of the cell volume), vs. the 10% of cellular volume typically ascribed to the ER (Alberts et al., 2014). The further concentration of AMIGO-1 in high density clusters, which we estimate comprise < 20% of the PM area of a neocortical cell body, would further contribute to the robust AMIGO-1 immunolabeling signal from PM clustered AMIGO-1 as present in sections from WT mice relative to signal from predominantly intracellular AMIGO-1 present in dKO mouse sections.

The relative impact of eliminating expression of Kv2.1 and Kv2.2 in the single and double KOs on the expression levels of AMIGO-1 may also provide valuable insights into the relative expression levels of these Kv2 α subunits, information not available from Kv2.1- and Kv2.2-specific immunolabeling. If we make the assumptions that AMIGO-1 is present at the same stoichiometry in all Kv2-containing channels, regardless of the proportion of Kv2.1 vs. Kv2.2 in these complexes, and that AMIGO-1 expression is similarly impacted by loss of Kv2.1 and Kv2.2. we can then use the relative impact of elimination of these Kv2 α subunits to estimate the relative expression levels of Kv2.1 and Kv2.2 in brain. As shown in Figure 11, elimination of Kv2.1 expression yields a larger (46%) reduction in AMIGO-1 expression than does elimination of Kv2.2 (9% reduction), suggesting that in mouse brain Kv2.1 may be ≈5X as abundant as Kv2.2. Note that while there are many caveats to this admittedly simple analysis, such differences in overall expression levels might contribute to the more robust overall behavioral phenotype observed in Kv2.1 KO mice (Speca et al., 2014) relative to more subtle behavioral phenotype reported for Kv2.2 KO mice (Hermanstyne et al., 2013).

We found that AMIGO-1 colocalizes with both Kv2.1 and Kv2.2 in mammalian brain neurons and in HEK293 cells, and that coexpression with either Kv2 α subunit is necessary for efficient cell surface expression and clustering of AMIGO-1. While Kv2 α subunits are required for the clustered somatodendritic localization of AMIGO-1 seen in brain neurons, AMIGO-1 does not reciprocally impact Kv2 α subunit clustering, as supported by our studies in heterologous cells, and by a recent report that showed that while a reduction in overall Kv2.1 expression levels was seen in AMIGO-1 KO mice, the remaining Kv2.1 remained localized in PM clusters on neuronal somata and proximal dendrites (Peltola et al., 2016). Our immuno-electron microscopy results show that AMIGO-1 is localized in the PM near the edges of the hypolemmal subsurface cisternae, similar to what has been previously demonstrated for Kv2.1 (Du et al., 1998; Mandikian et al., 2014) and Kv2.2 (Bishop et al., 2015), and our live cell imaging studies show that AMIGO-1:Kv2 complexes can recruit ER to ER:PM junctions. Interestingly, Kv2.1 alone also induces formation of ER:PM junctions (Antonucci et al., 2001; Cobb et al., 2015; Fox et al., 2015). Kv2.1 clusters in the PM of brain neurons are also closely opposed to astrocytic processes (Du et al., 1998), possibly to allow rapid removal of K^+^ from the extracellular space after K^+^ efflux through Kv2.1 channels. Moreover, Kv2.1 is functionally regulated by extrasynaptic glutamate (Misonou et al., 2008; Mulholland et al., 2008), whose levels are regulated in part by astrocyte glutamate uptake. The membrane topologies of Kv2 α subunits and AMIGO-1 are quite complementary, in that the extracellular domains of Kv2.1 and Kv2.2 are very limited compared their extensive cytoplasmic C- and N-terminal domains (comprising three-fourths of their entire primary structure), and which contain signals for clustering, and association with ER:PM junctions, while AMIGO-1 has a relatively small cytoplasmic C-terminal domain, and a large extracellular domain. It is interesting to speculate that the cell adhesion functions of AMIGO-1 may mediate the specific association of astrocyte processes with sites of neuronal Kv2 channel clustering (Du et al., 1998).

It is clear from our studies here that both in brain neurons *in vivo*, and in heterologous cells, AMIGO-1 clustering at sites overlaying intracellular hypolemmal subsurface cisternae and apposed to extracellular astrocytic processes requires Kv2 α subunit coexpression, consistent with the presence of an intrinsic clustering motif on Kv2.1 and Kv2.2 (Scannevin et al., 1996; Lim et al., 2000; Mohapatra and Trimmer, 2006; Bishop et al., 2015). It is intriguing that the Kv2.1 and Kv2.2 α subunit clustering mutants are still able to promote AMIGO-1 trafficking to the PM, and to enhance AMIGO-1 posttranslational processing, showing that clustering *per se* is not involved in the association of Kv2 α subunits and AMIGO-1, or in the Kv2-mediated enhancement of the PM trafficking of AMIGO-1. Structural information on the Kv2:AMIGO-1 complex itself will shed light on the details of their interaction, how AMIGO-1 influences Kv2 channel function and how Kv2 association profoundly impacts the expression and trafficking of AMIGO-1, as well as its function as a cell adhesion molecule.

## Author contributions

HB, MC, MK, LP, DM, AP, RS, KM, and JT: designed research; HB, MC, MK, LP, DM, AP, and MM: performed research; JK-P, HR, and JT: contributed unpublished reagents/analytic tools; HB, MC, MK, LP, DM, AP, RS, KM, and JT: analyzed data; HB, MC, LP, RS, KM, and JT: wrote the paper.

### Conflict of interest statement

The authors declare that the research was conducted in the absence of any commercial or financial relationships that could be construed as a potential conflict of interest.

## Supplementary material

Figure S1 Original immunoblot used as the source for the representative immunoblots shown in Figure 10A. Representative immunoblot of HEK293 cell lysates expressing AMIGO-1, AMIGO-1 + Kv2.1, AMIGO-1 + Kv2.2, Kv2.1, or Kv2.2. The leftmost lane is prestained molecular weight standards, only some of which show up in fluorescence. The respective expression patterns and the colors of the immunosignals are depicted in the label.

Figure S2 Original immunoblot used as the source for the representative immunoblot shown in Figure 10B. Representative immunoblot of AMIGO-1 expressing HEK293 cells treated with or without proteinase K (PK). The leftmost lane is prestained molecular weight standards, only some of which show up in fluorescence. The label depicts presence/absence of PK.

Figure S3 Original immunoblot used as the source for the representative immunoblot shown in Figure 11A. Representative immunoblot of crude whole brain homogenates from WT, Kv2.1 KO, Kv2.2 KO, and Kv2 double KO mice. Immunoblots were probed with mAbs against Kv2.1 (K89/34 mAb, green), Kv2.2 (N372B/60 mAb, red), AMIGO-1 (AMIGO-1 pAb, red), and Grp75 (N52A/42 mAb, green) as a loading control. The leftmost lane is prestained molecular weight standards, only some of which show up in fluorescence.
