# Supplementary figures and images for: Kv2 Ion Channels Determine the Expression and Localization of the Associated AMIGO-1 Cell Adhesion Molecule in Adult Brain Neurons
Source: Front Mol Neurosci. 2018 Jan 19;11:1. doi: 10.3389/fnmol.2018.00001 (PMC5780429; doi:10.3389/fnmol.2018.00001)

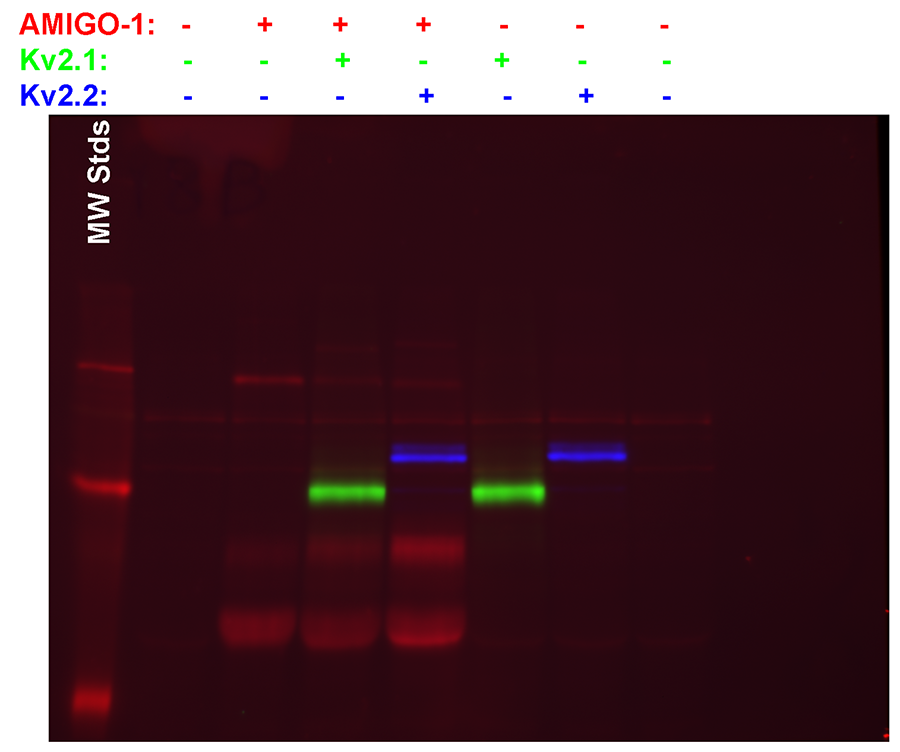

Supplement: Figure S1 — Original immunoblot used as the source for the representative immunoblots shown in Figure 10A. Representative immunoblot of HEK293 cell lysates expressing AMIGO-1, AMIGO-1 + Kv2.1, AMIGO-1 + Kv2.2, Kv2.1, or Kv2.2. The leftmost lane is prestained molecular weight standards, only some of which show up in fluorescence. The respective expression patterns and the colors of the immunosignals are depicted in the label. [file Image1.TIF]

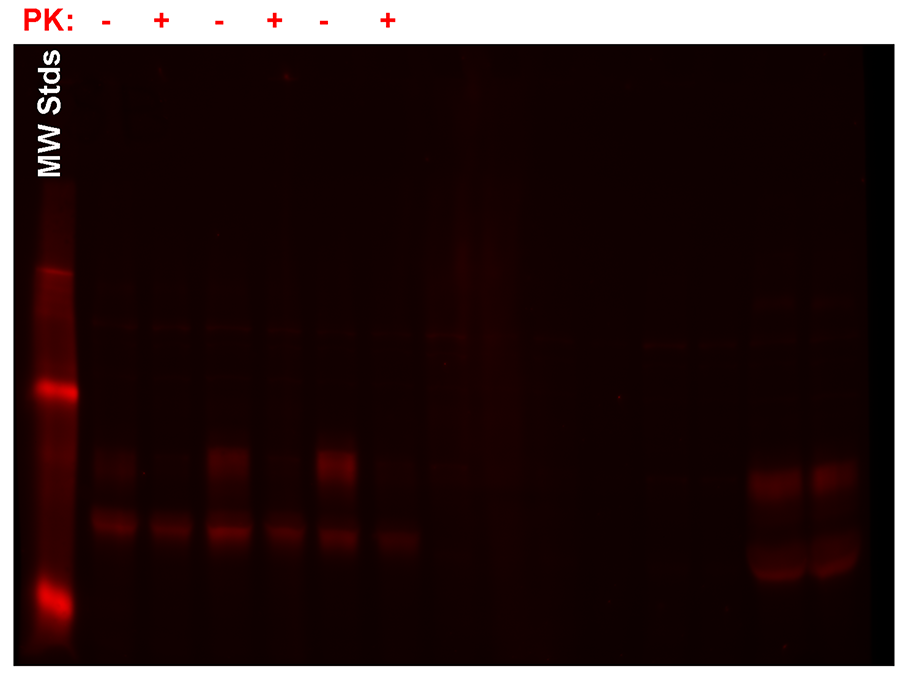

Supplement: Figure S2 — Original immunoblot used as the source for the representative immunoblot shown in Figure 10B. Representative immunoblot of AMIGO-1 expressing HEK293 cells treated with or without proteinase K (PK). The leftmost lane is prestained molecular weight standards, only some of which show up in fluorescence. The label depicts presence/absence of PK. [file Image2.TIF]

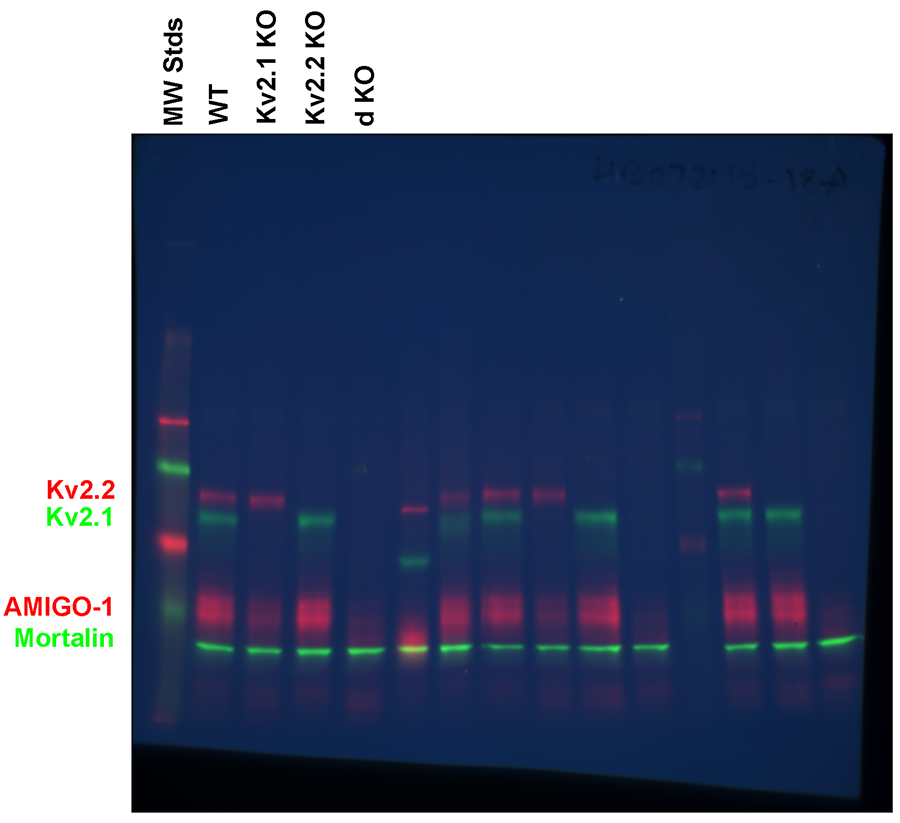

Supplement: Figure S3 — Original immunoblot used as the source for the representative immunoblot shown in Figure 11A. Representative immunoblot of crude whole brain homogenates from WT, Kv2.1 KO, Kv2.2 KO, and Kv2 double KO mice. Immunoblots were probed with mAbs against Kv2.1 (K89/34 mAb, green), Kv2.2 (N372B/60 mAb, red), AMIGO-1 (AMIGO-1 pAb, red), and Grp75 (N52A/42 mAb, green) as a loading control. The leftmost lane is prestained molecular weight standards, only some of which show up in fluorescence. [file Image3.TIF]
